# APOE Stabilization by Exercise Prevents Aging Neurovascular Dysfunction and Complement Induction

**DOI:** 10.1371/journal.pbio.1002279

**Published:** 2015-10-29

**Authors:** Ileana Soto, Leah C. Graham, Hannah J. Richter, Stephen N. Simeone, Jake E. Radell, Weronika Grabowska, W. Keith Funkhouser, Megan C. Howell, Gareth R. Howell

**Affiliations:** 1 The Jackson Laboratory, Bar Harbor, Maine, United States of America; 2 Sackler School of Graduate Biomedical Sciences, Tufts University, Boston, Massachusetts, United States of America; Baylor College of Medicine, UNITED STATES

## Abstract

Aging is the major risk factor for neurodegenerative diseases such as Alzheimer's disease, but little is known about the processes that lead to age-related decline of brain structures and function. Here we use RNA-seq in combination with high resolution histological analyses to show that aging leads to a significant deterioration of neurovascular structures including basement membrane reduction, pericyte loss, and astrocyte dysfunction. Neurovascular decline was sufficient to cause vascular leakage and correlated strongly with an increase in neuroinflammation including up-regulation of complement component C1QA in microglia/monocytes. Importantly, long-term aerobic exercise from midlife to old age prevented this age-related neurovascular decline, reduced C1QA+ microglia/monocytes, and increased synaptic plasticity and overall behavioral capabilities of aged mice. Concomitant with age-related neurovascular decline and complement activation, astrocytic *Apoe* dramatically decreased in aged mice, a decrease that was prevented by exercise. Given the role of APOE in maintaining the neurovascular unit and as an anti-inflammatory molecule, this suggests a possible link between astrocytic *Apoe*, age-related neurovascular dysfunction and microglia/monocyte activation. To test this, *Apoe*-deficient mice were exercised from midlife to old age and in contrast to wild-type (*Apoe*-sufficient) mice, exercise had little to no effect on age-related neurovascular decline or microglia/monocyte activation in the absence of APOE. Collectively, our data shows that neurovascular structures decline with age, a process that we propose to be intimately linked to complement activation in microglia/monocytes. Exercise prevents these changes, but not in the absence of APOE, opening up new avenues for understanding the complex interactions between neurovascular and neuroinflammatory responses in aging and neurodegenerative diseases such as Alzheimer’s disease.

## Abstract 2

Ileana Soto, Gareth Howell, and coauthors find that age-related deterioration of the neurovascular unit and increased neuroinflammation in aging mice is prevented by long-term exercise, but not in the absence of apolipoprotein E.

## Author Summary

Aging is frequently accompanied with frailty and cognitive decline. In recent years, increasing evidence has linked physical inactivity with the development of dementias such as Alzheimer’s disease. In fact, it is recognized that exercise combats frailty and cognitive decline in older adults, but the biological mechanisms involved are not completely known. Understanding the biological changes that trigger cognitive deterioration during aging and the mechanisms by which exercise improves health and brain function is key to ensuring the quality of life of the elderly population and to reducing risk of dementias such as Alzheimer’s disease. Here, we show that the cerebrovascular system in mice significantly deteriorates with age, and the structure and function of the blood brain barrier is progressively compromised. These age-related neurovascular changes are accompanied by neuroinflammation and deficits in common and spontaneous behaviors in mice. We found, however, that exercise from middle to older age preserves the cerebrovascular health, prevents behavioral deficits and reduces the age-related neuroinflammation in the cortex and hippocampus in aged mice. Mice deficient in *Apoe*, a gene associated with longevity and Alzheimer’s disease, are resistant to the beneficial effects of exercise, suggesting a possible mediating role for APOE in the maintenance and function of the neurovascular system during aging.

## Introduction

As the general population ages, age-related diseases are on the increase. Data from the United States (US) Department of Health and Human Services show that more than 12.9% of the US population is over the age of 65 and this is expected to double by 2030 [1]. Aging is the major risk factor for many diseases including cancer, diabetes, heart disease, and Alzheimer’s disease (AD) [2]. Therefore, with an aging population, prevalence of age-related diseases is expected to increase. For instance, more than 5 million people in the US suffer from AD, and this is expected to exceed 10 million in the next 20 years [3]. In order to prevent and treat age-related neurodegenerative diseases, particularly AD, it is essential to better understand the factors that contribute to aging-induced susceptibility.

In the healthy aged brain, functional and morphological changes lead to cognitive and sensorimotor control decline that affect the performance of activities of daily living (ADL) in older adults and increase vulnerability to the development of neurodegenerative conditions [4,5]. Age-related cognitive deficits are partly explained by changes in neural plasticity and synaptic activity. However, overall decline in brain health has also been correlated to other non-neuronal processes such as cerebrovascular dysfunction [6,7] and activation of innate immune responses [8]. Understanding how aging affects these processes will likely shed light on developing therapeutic strategies that prevent these age-related changes and decrease the vulnerability to neurodegenerative conditions.

Interestingly, physical activity during aging has ameliorative effects on cognitive decline and sensorimotor deficits. Physically active older adults show improved performance in cognitive and sensorimotor tests and have greater brain volume in regions noticeably affected by age in sedentary subjects [4,9]. For instance, in humans, exercise enhances cerebral blood flow (CBF), neurogenesis, and angiogenesis in the hippocampal dentate gyrus, increases hippocampal volume, and improves memory [10,11,12]. In addition, aerobic physical activity greatly improves cardiovascular function and decreases systemic inflammation in older subjects [8,9,13]. However, a detailed analysis of the processes involved has not been performed. In this study, we demonstrate significant changes in neurovascular integrity and function in the superior region of the cortex and hippocampus. We show a significant loss of pericytes and marked neurovascular decline, correlated with dysfunction of the blood brain barrier and activation of innate immune responses including the complement cascade. Strikingly, exercise almost completely prevented these age-related neurovascular changes and lessened complement induction in myeloid cells, but had little to no effect in the absence of APOE. We propose that exercise is an effective means of mitigating age-related neurovascular decline by directly or indirectly modulating *Apoe*-expressing astrocytes and/or C1QA-expressing myeloid cells.

## Results

### Gene Profiling Suggests Aging Causes Regional Compromise of the Neurovascular Unit

To determine gene expression changes in response to normal aging, transcriptional profiling was performed on brain tissue from young (4 mo) and aged (21 mo) C57BL/6J (B6) mice. Because portions of the limbic and higher-order integrative cortical areas (including frontal association cortex, cingulate cortex, retrosplenial cortex, and the parietal associative cortices) and the hippocampus are commonly impacted in AD [14], brains were dissected into three separate regions for RNA sequencing and analysis: (i) region 1—frontoparietal cortex and corpus callosum (FPC/CC), (ii) region 2—hippocampus (HP), and (iii) region 3—rest of the cortex and brain stem (RB). In total, 24 samples were profiled separately—three regions from four different mice from two age groups. To avoid batch effects, all RNA was prepared, and RNA-seq libraries generated at the same time. Samples were barcoded, pooled and sequenced across four lanes of an Illumina Hi-seq (see Methods).

**Fig 1:**
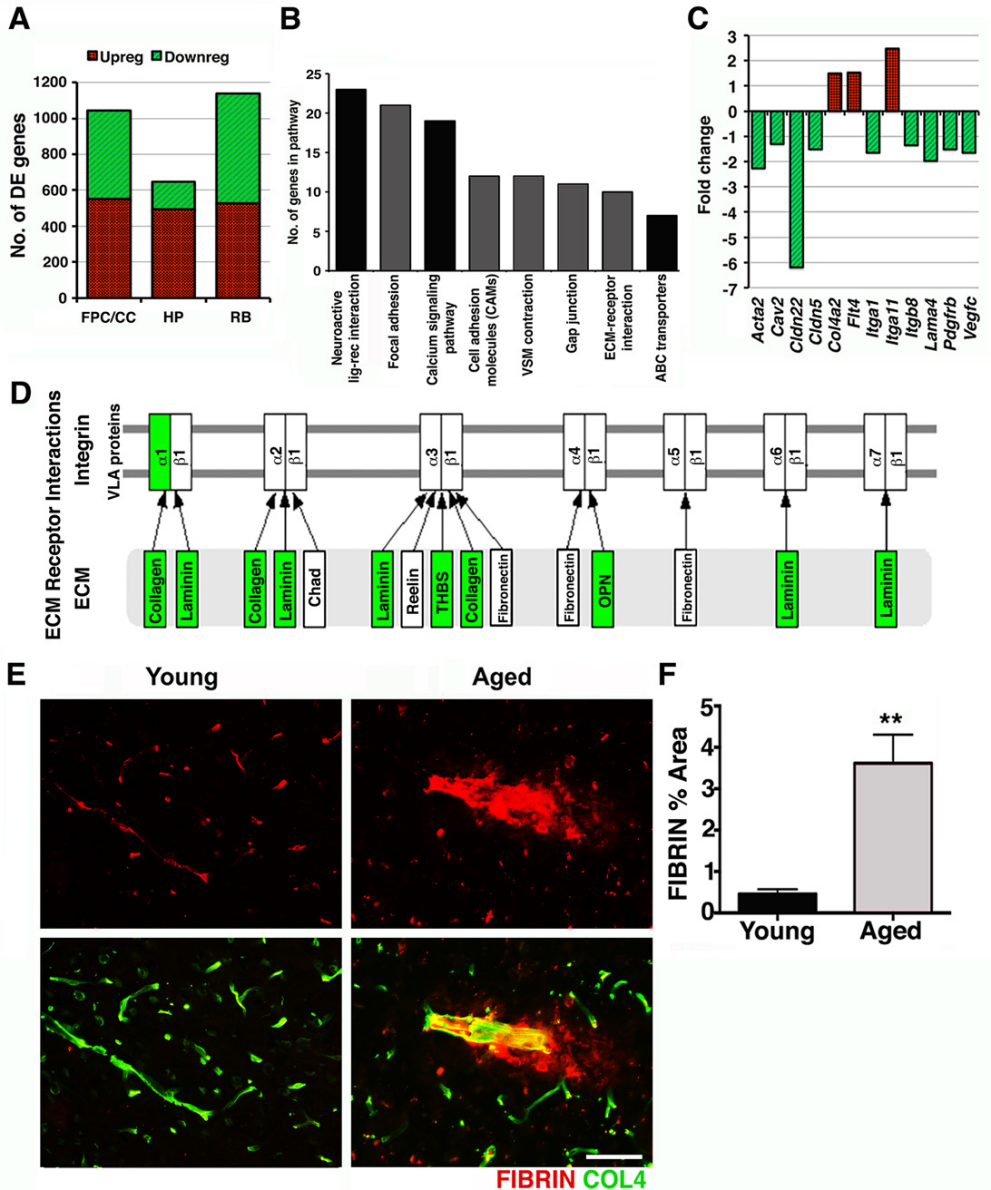
Transcriptional profiling predicts age-related neurovascular dysfunction. (A) The number of DE genes in three brain regions (FPC/CC; HP; RB) comparing 21-month-old mice to 4-month-old control mice. (B) Pathways overrepresented in the DE genes from the FPC/CC region. For further details and the overrepresented pathways from other regions, see S4 Table. (C) Genes relevant to the neurovascular unit were generally down-regulated. (D) DE genes from the FPC/CC region in the ECM-receptor interaction pathway were down-regulated (green). (E–F) Fibrin intra- and extravascular deposits (red) were significantly increased in aged cortex compared to young cortex. In (**F**) values are relative mean + Standard Error of the Mean (SEM) to the young values, *n* = 6 mice per group, **p = 0.0073 by unpaired *t* test. Scale Bars: 50 μm. The data used to make this figure can be found in S1 Dataset.

For each of the three regions profiled, pairwise analyses comparing young samples to aged samples were performed to determine differentially expressed (DE) genes (see Methods). A total of 1,045 genes (551 up-regulated and 494 down-regulated) were DE in the FPC/CC (region 1, S1 Table), 644 genes (492 up-regulated and 152 down-regulated) in the HP (region 2, S2 Table), and 1,137 (526 up-regulated and 611 downregulated) in the RB (region 3, S3 Table) (Fig 1A). Gene set enrichment analysis (using DAVID, see Methods) showed significant differences between the regions, i.e., DE genes from each of the three regions were present in overlapping but not identical Kyoto Encyclopedia of genes and genomes (KEGG) pathways (S4 Table). This suggests that normal aging impacts the regions of the brain in different ways and may provide clues as to why specific brain regions are more susceptible to dysfunction in certain neurodegenerative diseases. Of particular interest were the KEGG pathways overrepresented in region 1, tissue enriched for the FPC and the CC. These pathways included focal adhesion, vascular smooth muscle contraction, gap junction and extracellular matrix (ECM)-receptor interaction pathways (Fig 1B). Genes in these pathways were generally down-regulated (Fig 1C and 1D) and suggest possible perturbation to the neurovascular unit. To assess possible neurovascular dysfunction in region 1, intravascular and perivascular deposition of the plasma protein fibrinogen, or fibrin, a marker of vascular compromise, was analyzed by immunostaining. Previous studies have shown intra- and extravascular accumulation of fibrin(ogen) in the postmortem brains of AD patients as well as AD mouse models where severe neurovascular dysfunction occur [15,16,17,18]. In aged mice, small and sporadic deposits of extravascular fibrin were found only in the cortex, particularly the neocortex (region 1) (Fig 1E). We also noticed that contrary to young mice, intravascular accumulation of fibrin(ogen) was persistent in the brains of aging mice even after intracardial perfusion suggesting possible deposition of fibrin(ogen) in the luminal vessel wall as previously observed in human AD brains [15]. Quantification of fibrin(ogen)-immunostained area in the cortex, which included intra- and extravascular deposition of this protein, indicated a 3-fold increase in aged compared with young mice (Fig 1F), confirming the transcriptional profiling data that predicted neurovascular dysfunction in the FPC in aged mice.

### Aging Causes BM Breakdown and Pericyte Loss

**Fig 2:**
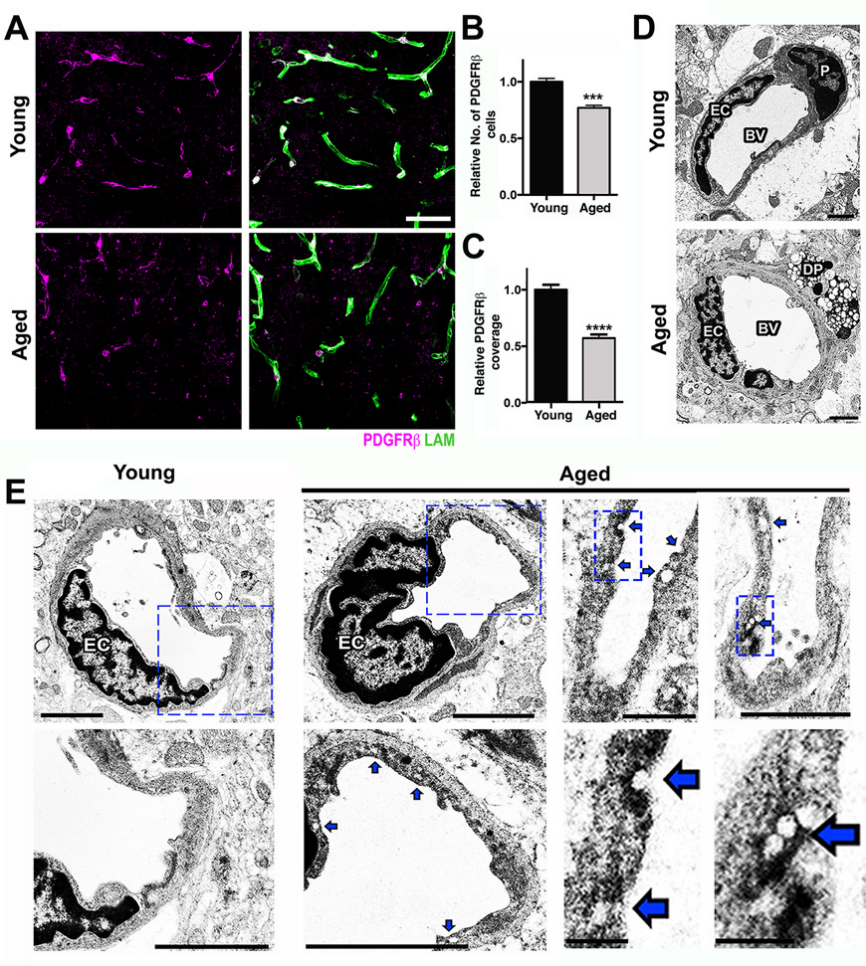
Pericyte loss and increased endothelial transcytosis in the aged cortex. (A) Reduction of PDGFRβ^+^ pericytes (magenta) and laminin capillary coverage (green) is evident in the aged cortex when compared with the young cortex. (B) Quantification of PDGFRβ^+^ pericytes in the young and aged cortex. (C) Quantification of PDGFRβ^+^ pericyte coverage of microvessels in the young and aged cortex. (D) In the young cortex, a healthy pericyte (P) is shown, while in the aged cortex, a degenerating pericyte (DP) and thicker BM are displayed. (E) Ultrastructural analysis of young and aged blood vessels in the cortex reveals increased number of vesicles and indentations (blue arrows) in the endothelial cells from aged mice, suggesting an increase in vesicular transcytosis activity in these cells during aging. In B and C, values are relative mean + SEM to the young values, *n* = 6 mice per group, ***p < 0.001 and ****p < 0.0001 by unpaired *t* test. Scale Bars: 50 μm (A), 2 μm (D and E), and 0.5 μm and 0.25 μm (E two lower panels at the right). EC = endothelial cell and BV = blood vessel. The data used to make this figure can be found in S1 Dataset.

The transcriptional profiling of the aged FPC/CC demonstrated significant down-regulation of extracellular matrix-associated genes as well as important genes for pericyte function suggesting possible disturbances in the interactions and function of the components of the neurovascular unit. To more fully characterize the extent of neurovascular dysfunction in the cortex of aged brains, the major components of the neurovascular unit, including basement membrane (BM), endothelial cells, pericytes, and astrocytes were assessed. Collagen IV (COL4) and laminin (LAM), two major components of the BM (generated by astrocytes, pericytes, and endothelial cells), were significantly reduced in the cortex of aged mice compared to young mice (S1 and S2 Figs) despite no decline in CD31, a marker of endothelial cells (S1 Fig). This suggests BM reduction was not caused by vascular reduction due to endothelial cell loss. Gene profiling showed three pericyte-related genes, *Pdgfrβ*, *Act2*, and *Cav2*, which were significantly down-regulated in aged compared to young mice (Fig 1C), suggesting pericytes were negatively impacted by normal aging. Analysis of pericytes, using immunostaining for PDGFRβ showed a greater than 20% reduction in pericyte numbers and almost 50% reduction in pericyte coverage of microvessels in the cortex of aged compared to young brains (Fig 2A–2C).

**Fig 3:**
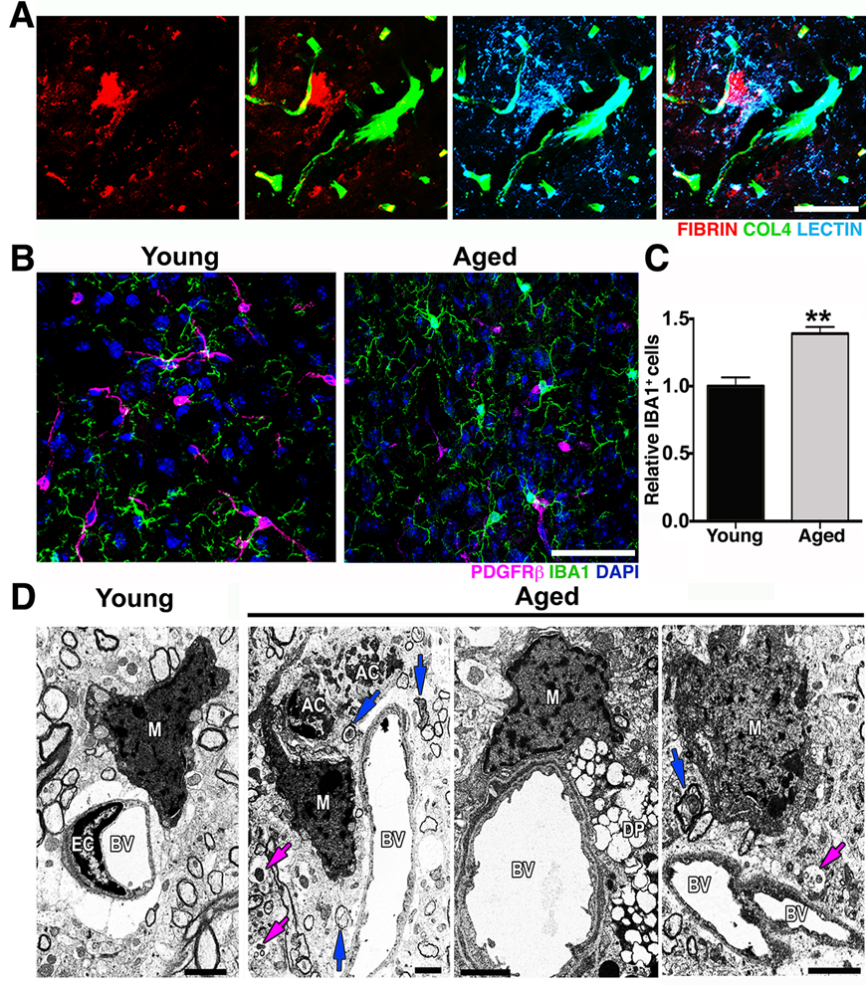
Microglia/monocyte density is increased in regions with pericyte loss in the aged cortex. (A) Representative images showing extravascular deposition of FIBRIN (red) surrounded by microglia with increased LECTIN immunoreactivity (light blue). (B–C) A significant increase in IBA1^+^ microglia/monocyte cells (green) in the aged cortex coincides with the significant decrease of PDGFRβ^+^ pericytes (magenta) in the same region when compared with the cortex from young mice. (D) In the aged cortex, electron micrographs show active phagocytic microglia/monocytes (M) in close proximity to apoptotic cell bodies (AC) in close contiguity to a blood vessel (BV), to a degenerated pericyte (DP), and to a blood vessel with high transcytosis activity. Damaged axons (blue arrows) and vesicular bodies with cellular debris (magenta arrows) are also observed in these regions. In (C) values are relative mean + SEM to the young values, *n* = 4 mice per group, **p < 0.005 by unpaired *t* test. Scale Bars: 50 μm (A–B) and 2 μm (D). The data used to make this figure can be found in S1 Dataset.

Although transcriptional profiling data in the HP did not show pathways relevant to neurovascular unit dysfunction as being differentially affected by age (S4 Table), genes relevant to the neurovascular unit, including *Pdgfrβ* (−1.36) and *Cldn5* (−1.63) were down-regulated (S2 Table), suggesting possible dysfunction to the neurovascular unit in the HP also. Therefore, we assessed neurovascular health in the CA1 region of the HP. Similar to our findings in the FPC, COL4^+^ microvessels and coverage of PDGFRβ^+^ were also significantly reduced in the hippocampal CA1 region of aged mice when compared with young (S3 Fig). Furthermore, ultrastructural analysis identified several degenerating pericytes in the cortex of aged mice that were not observed in young mice (Fig 2D). Pericytes are critical components of the neurovascular unit and play a key role in the regulation of BM, vessel contractility, and inhibition of vesicular transcytosis through endothelial cells [19]. Increased endothelial vesicular transcytosis was frequently observed in the aged cortex (Fig 2E), further supporting age-related pericyte dysfunction or loss. Fibrin deposition and pericyte loss were associated with an increase in the number of activated microglia/monocytes (Fig 3A and 3B). Ultrastructural analyses suggested that these microglia/monocytes were phagocytic when in close proximity to areas of pericyte degeneration and increased endothelial transcytosis activity (Fig 3C).

**Fig 4:**
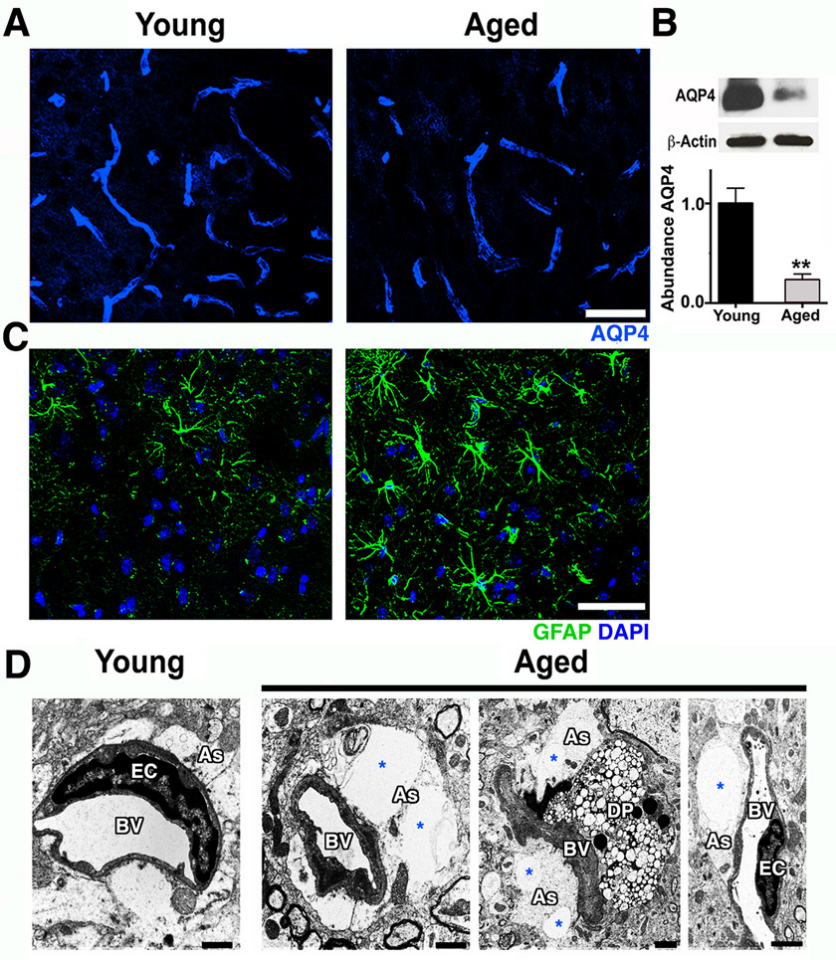
Astrocytic AQP4 is decreased in aged cortical astrocytes. (A) AQP4 immunoreactivity (blue) and protein levels (B) are significantly decreased in the aged cortex. (C) Astrocyte reactivity is increased in the cortex of aged mice determined by increased immunoreactivity of astrocytic GFAP (green) when compared with young mice. (D) Electron micrographs showing examples of astrocyte endfeet (As, white region surrounding the vessels) abnormalities such as swelling and big vacuoles (*) in aged mice. Astrocyte endfeet abnormalities were not observed in all cases in aged mice and never seen in young mice. Values in (B) are relative mean + SEM to the young values, *n* = 4 per group. **p < 0.005 (B) by unpaired *t* test. Scale Bars: 50 μm (A and C) and 2 μm (D). DP = degenerated pericyte, EC = endothelial cell and BV = blood vessel. The data used to make this figure can be found in S1 Dataset.

Pericytes can mediate the attachment of astrocyte endfeet to the vascular surface and regulate the polarization of specific proteins, such as the water channel aquaporin-4 (AQP4), to the perivascular endfoot region [20,21]. AQP4 is expressed by astrocytes that play a key role in the regulation of brain water transport at the neurovascular interface [22]. Immunostaining and immunoblotting showed a significant decrease of AQP4 protein at the neurovascular junctions in the cortex of aged compared to young mice (Fig 4A and 4B). AQP4 decrease was accompanied by an increase in glial fibrillary acidic protein (GFAP) immunoreactivity, an indication of astrocyte reactivity (Fig 4C). There was also a noticeable swelling of astrocyte endfeet with enlarged vacuoles in some cortical microvessels of aged mice but not from young mice (Fig 4D). Collectively, our data suggest that normal aging causes significant dysfunction to the cortical neurovascular unit, including BM reduction and pericyte loss. These changes correlate strongly with an increase in microglia/monocytes in the aged cortex.

### Exercise Prevents Neurovascular Dysfunction and Complement Induction in Aged Mice

**Fig 5:**
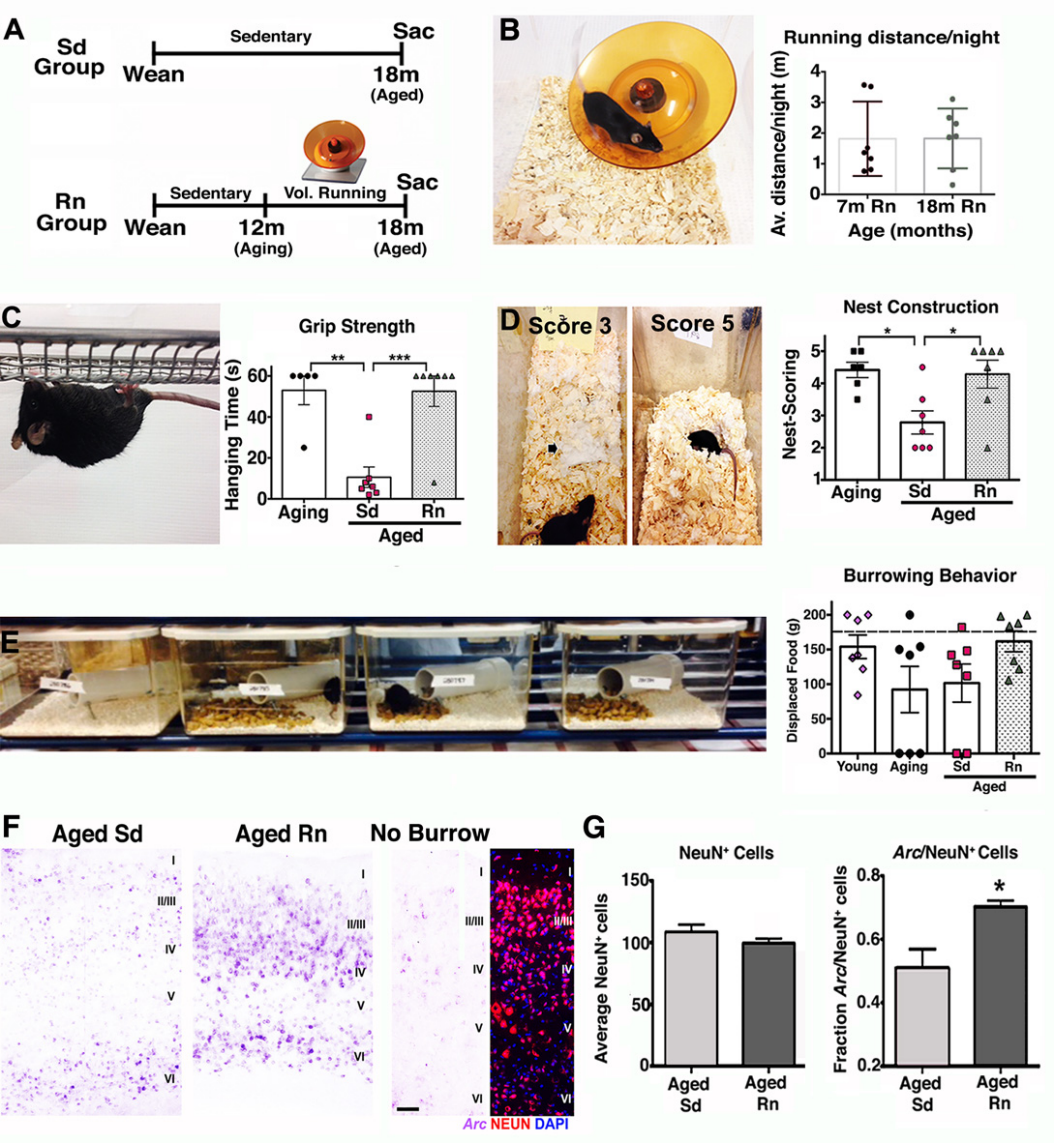
Exercise improves behavior and neuroplasticity in aged mice. (A) Experimental strategy for voluntary running experiments (Sd = sedentary, Rn = Runner, Sac = sacrifice). (B) Representative image of a mouse running in an electronic wheel and quantification of running distance/night for young and aged mice after 6 months showing no differences between groups. (C) Significant deficits in grip strength found in aged sedentary mice were prevented by voluntary running. (D) Representative images of nests scored 3 and 5 are shown. Nest construction behavior was preserved in aged running mice but not in aged sedentary mice. (E) No statistically significant changes were found in burrowing behavior, however mice in the aging group (3/7) and in the aged sedentary group (2/6) did not engage in this activity, while all young and runner aged mice were able to perform it. (F) *Arc* (purple) in situ hybridization in aged sedentary and aged runner mice after 2 h of burrowing test. Low levels of *Arc* expression are found in the cortex of a mouse that did not do the burrowing test. (G) The fraction of NeuN^+^ *Arc* ^*+*^ neurons is significantly higher in the aged runners when compared with aged sedentary mice. No changes in the number of cortical NeuN^+^ neurons between aged sedentary and running mice. In panels B–E and G, values are mean + SEM, *n* = 7 per group in B–E, and *n* = 5 per group in G. In (C) **p = 0.0012 ***p = 0.0006, in (D) *p = 0.0165 and 0.0217 respectively, and in (G) *p = 0.0282 by ANOVA followed by Tukey’s posthoc tests. Scale Bar: 50 μm. The data used to make this figure can be found in S1 Dataset.

Aging is generally accompanied by cognitive decline and sensorimotor deficits that affect the performance of ADL in the aged population [4,5]. Lifestyle choices such as exercise have been shown to have beneficial effects on the aging brain [9,13], including increased brain volume [4,11], improved performance in several cognitive and motor tasks [4], and neuronal function [9]. Also, a recent study claimed a third of AD cases could be attributed in part to physical inactivity [23]. However, the impact of long-term physical exercise on the health of the neurovascular unit has not been determined. To assess this, mice were provided access to a running wheel from 12 months old (equivalent to middle aged in humans) and assessed at 18 months of age (equivalent to early old age [~60 y old] in humans where risk of AD is greatly increased) (Fig 5A). Voluntary running was preferred to exercise by forced treadmill to remove any potential confounding effects of stress [24]. In addition, voluntary running in mice can induce adaptive physiological changes in cardiac and skeletal muscle showing it is a good method to assess biological changes as a result of exercise [25]. No differences in average running distance (~2 miles/night/mouse) between the young (7 mo) and aged (18 mo) groups of mice were found after 6 months with the running wheel (Fig 5B), indicating that aged mice were able to maintain their running capacity during the 6 months period. Exercised aged mice were first assessed for overall improvement in behavior and neuronal activity. Physical activity improves ADL in humans, and so common daily behaviors in mice—grip strength, nesting, and burrowing—were assessed in exercised and nonexercised (sedentary) mice (Fig 5C–5E). Significant improvements in both grip strength (Fig 5C) and nesting behavior (Fig 5D) were observed in running aged mice (18 mo) compared to sedentary aged mice (18 mo) and were similar to levels seen in aging (12 mo) mice. Burrowing also appeared to be improved but was not statistically significant (Fig 5E). These results indicate that physical activity improved the capabilities and motivation of old mice to engage and perform typical spontaneous behaviors that seem to be affected by aging.

These behavioral improvements induced by exercise were accompanied by stabilization of functional synapses and improved neural plasticity. While sedentary aged mice showed significant decreased levels of the presynaptic proteins such as synaptophysin and the postsynaptic protein PSD-95 (S4 Fig), indicating a possible loss or weakening of functional synapses, running mice demonstrated significant preservation of synaptophysin when compared with younger mice (S4 Fig). Neural plasticity was also evaluated in these mice by examining changes in the expression of the immediate early gene *Arc* (activity-regulated cytoskeletal gene). *Arc* transcription is induced by neuronal activity [26] and is immediately up-regulated in the parietal cortex in response to spatial exploration [27]. *Arc* cortical expression was significantly elevated in aged running mice compared with aged sedentary mice after burrowing behavior (Fig 5F and 5G), indicating that more neurons in the parietal cortex were recruited and activated during spatial exploration in the aged runner mice compared to aged sedentary mice.

**Fig 6:**
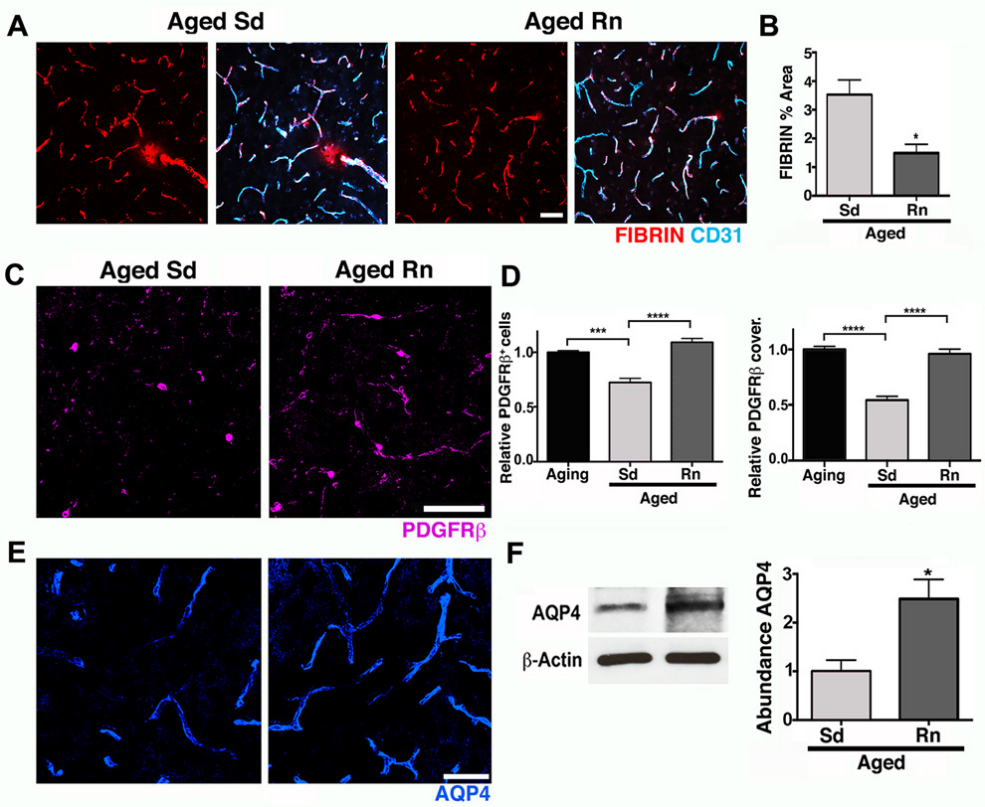
Exercise preserves neurovascular unit integrity in aged mice. (A–B) Age-related intravascular and extravascular deposition of FIBRIN (red) is significantly reduced in aged runner mice. Microvessels are immunostained with endothelial CD31 (cyan). (C) PDGFRβ^+^ pericyte (magenta) number and coverage are significantly increased in aged runners when compared with aged sedentary mice. (D) Quantitative analysis of PDGFRβ^+^ pericytes number and coverage. (E) AQP4 (blue) immunoreactivity of astrocyte endfeet is significantly increased in aged runners when compared with aged sedentary mice. (F) AQP4 protein levels show a significant preservation of both parameters on aged runner mice. In panels (D–F), values are mean + SEM, *n* = 4 aging mice, *n* = 6 aged Sd mice and *n* = 6 aged Rn mice. In (B) *p = 0.014 and (F) *p = 0.0318 by unpaired *t* test. In (D) ****p < 0.0001 and ***p = 0.0014 by ANOVA followed by Tukey’s posthoc tests. Scale Bars: 50 μm. The data used to make this figure can be found in S1 Dataset.

Next, the effects of exercise on the neurovascular unit were assessed. Exercise significantly reduced vascular leakage. Fibrin levels in aged running mice were similar to that seen in young mice and significantly less than aged sedentary mice (Fig 6A and 6B). COL4 immunostaining in the cortex (S5 Fig) and CA1 (S5 Fig) demonstrated a significant preservation of the BM in aged runner mice when compared with aged sedentary mice (S5 Fig). No changes were observed in the density of CD31^+^ microvessels between sedentary and runner mice (S4 Fig), indicating that exercise prevented a deterioration of COL4 coverage in cortical and hippocampal CA1 microvessels during aging without an overall increase in vascular density. Aged runner mice also exhibited significantly higher numbers and vascular coverage of cortical PDGFRβ^+^ pericytes compared to aged sedentary mice, levels similar to those observed in younger sedentary mice (Fig 6C and 6D). Finally, exercise preserved astrocytic AQP4 protein to similar levels observed in young mice (Fig 6E and 6F) and correlated with a decrease in astrocyte reactivity (S6 Fig). In the hippocampal CA1 region, the reduction in vascular coverage of PDGFRβ^+^ pericytes and AQP4 levels observed in the aged sedentary mice were also prevented by exercise (S7 Fig). Therefore, exercise prevented age-related pericyte loss, neurovascular unit decline, and vascular leakage.

**Fig 7:**
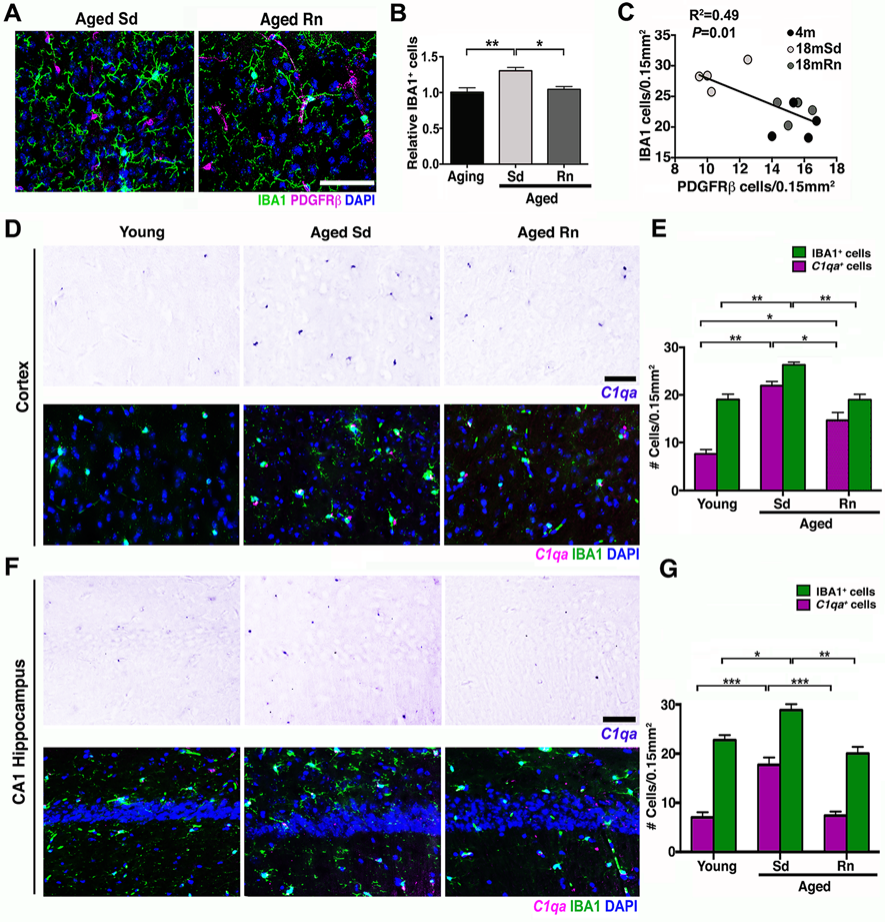
Exercise reduces *C1qa* ^+^ microglia/monocytes in the aged cortex. (A) Representative sections from an aged sedentary and aged runner mouse immunostained with PDGFRβ (magenta) for pericytes and IBA1 (green) for microglia/monocyte cells. (B) Quantification of IBA1^+^ cells in aging, aged sedentary (Sd), and aged runner (Rn) mice shows a decrease in microglia/monocyte density in the aged runners versus the aged sedentary mice. (C) Correlation between PDGFRβ^+^ cells and IBA1^+^ cells (R^2^ = 0.49, *p* = 0.01). Decreased number of pericytes correlates with increasing number of microglia/monocyte cells. (D) Representative sections of the cortex hybridized with *C1qa* riboprobe and coimmunostained with IBA1 (green) in young, aged Sd and aged Rn mice. (E) Quantification of *C1qa*/IBA1^+^ (magenta bars) and IBA1^+^ (green bars) cells in the cortex of young, aged Sd, and aged Rn mice showing a significant increase in *C1qa* ^+^/IBA1 positive cells in aged Sd mice and a significant decline of these cells in the aged runner mice. A new quantification of IBA1^+^ cells (different from B) was performed for the analysis of *C1qa* ^+^/IBA1 cells. (F) Representative sections of the hippocampal CA1 hybridized with *C1qa* riboprobe and coimmunostained with IBA1 (green) in young, aged Sd, and aged Rn mice. (G) *C1q*/IBA1^+^ (magenta bars) and IBA1^+^ (green bars) cells in the hippocampal CA1 are significantly increased in aged Sd mice and significantly reduced in aged Rn mice. In panels (B, E, and G) values are mean + SEM, *n =* 4 per group. In (B) **p = 0.0032 and *p = 0.0148, in (E) **p = 0.0065 and 0.0044, ***p = 0.0006 and *p = 0.0194 and in (G) ***p < 0.0006,***p = 0.0003, **p = 0.0025 and *p = 0.0302 by ANOVA followed by Tukey’s posthoc tests. Scale Bars: 50 μm. The data used to make this figure can be found in S1 Dataset.

Induction of the complement cascade in the brain, particularly in myeloid-derived cells such as microglia/monocytes, has been shown to be a potentially damaging event in aging and disease [28,29,30,31]. Further, the classical pathway of the complement cascade has been strongly implicated in synaptic and neuronal dysfunction [32], but only limited data are available on the role of complement in neurovascular dysfunction [33]. Analysis of our transcriptional profiling showed increased expression of multiple components of the classical pathway including *C1qa* (+1.94), *C1qb* (+1.91), *C1qc* (+2.02), *C3* (+2.71), *C4a* (+2.42), *C4b* (+4.63), and *C5ar2* (+2.81) in the HP of aged compared to young mice (see S2 Table). Since exercise prevented neurovascular dysfunction, synaptic decline, and behavioral deficits, we first assessed the impact of exercise on the number of microglia/monocytes in aged brains. Exercise significantly reduced the numbers of IBA1^+^ microglia/monocytes in the cortex of aged running mice compared to sedentary mice (Fig 7A and 7B). In fact, the numbers of microglia/monocytes were inversely correlated with pericyte numbers (Fig 7C), suggesting an important link between pericyte loss (and neurovascular health) and activation of microglia/monocytes. Next, to examine the effects of exercise on induction of the classical pathway of the complement cascade, we assessed *C1qa*, a component of the C1 complex, the initiating complex of the classical pathway. The number of *C1qa* ^*+*^ microglia/monocytes was elevated 3-fold in the aged mice compared to young mice (Fig 7D and 7E). Importantly, exercise caused a significant reduction in the number of cortical *C1qa* ^*+*^ microglia/monocytes (35% less, Fig 7E). Similarly, in the hippocampal CA1 region, microglia/monocytes were increased in aged sedentary mice when compared with young mice, and this increase was prevented by exercise in aged running mice (Fig 7F and 7G). These results suggest that the positive effects of exercise could be due in part to a reduction in complement activation during aging.

### Exercise Had No Effect on Behavioral Deficits, Neurovascular Dysfunction, and Microglial/Monocyte Activation in *Apoe*-Deficient Mice

**Fig 8:**
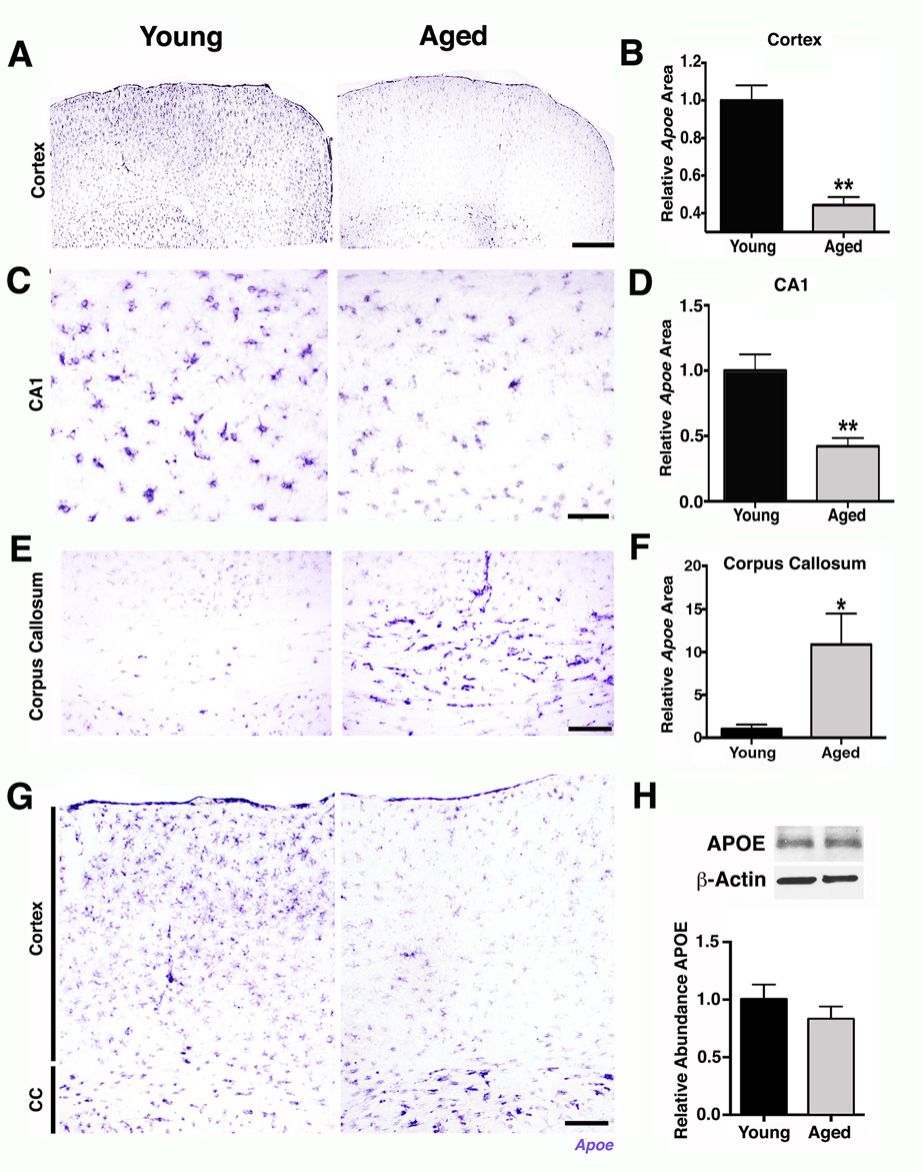
*Apoe* expression is reduced in the parietal cortex and CA1 of aged mice. (A) Representative sections of the FPC from young and aged brains hybridized with an *Apoe* riboprobe. (B) Quantification of *Apoe* hybridized area in the cortex shows a significant decrease of *Apoe* expression in the aged mice. (C) Representative sections of the hippocampal CA1 region from young and aged brains hybridized with an *Apoe* riboprobe. (D) *Apoe* hybridized area in the CA1 shows a significant decrease of *Apoe* expression in the aged mice. (E) Representative sections of the CC from young and aged brains hybridized with *Apoe* riboprobe. (F) A significant increase of *Apoe* hybridized area is measured on the aged CC. (G) Representative images of *Apoe* in situ hybridization in the cortex and CC showing decreased expression of *Apoe* in the cortex and increased expression in the CC in aged mice compared with young mice. (H) No differences in APOE protein levels were found by western blotting of whole brains from young and aged mice. Values in (B, D, and F) are relative mean + SEM to the young values, *n* = 6 per group. In (D) **p < 0.001 and in (F) *p = 0.0364 by unpaired *t* test. Scale Bars: 600 μm (A), 50 μm (C), 100 μm (E and G). The data used to make this figure can be found in S1 Dataset.

**Fig 9:**
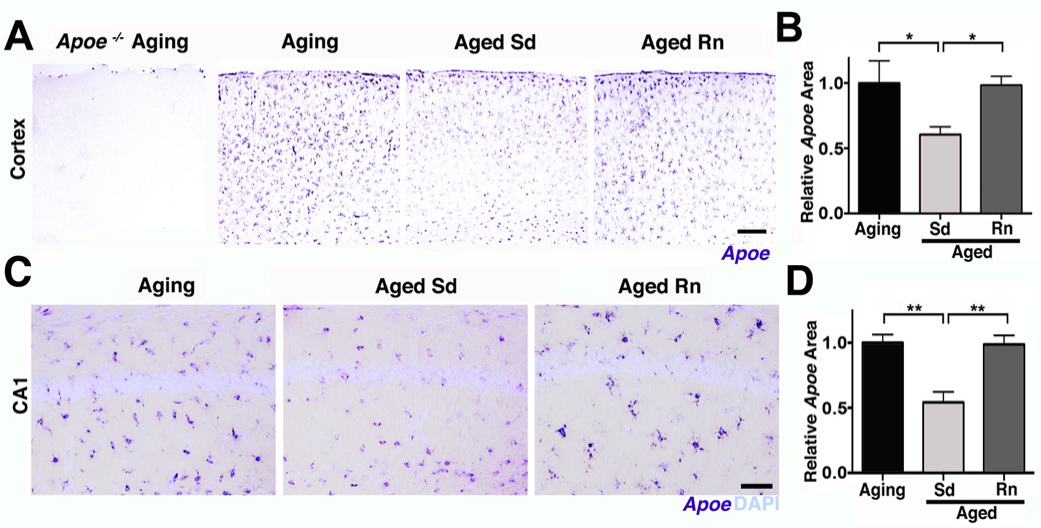
Age-related reduction in *Apoe* expression in the cortex and CA1 is prevented by running. (A–B) Representative sections and quantitative analysis of *Apoe*-hybridized cortex on aging *Apoe* ^*-/-*^, aging, aged sedentary (Sd), and aged runner (Rn) mice showing a significant preservation of *Apoe* expression on runner mice. No hybridization signal with *Apoe* riboprobe was observed in the *Apoe* ^*-/-*^ mouse brain. (C–D) Representative sections and quantitative analysis of *Apoe* expression on aging hippocampal CA1. Down-regulation of *Apoe* expression in the CA1 region in aging mice is prevented by wheel running. Values in (B and D) are relative mean + SEM to the aging values, *n* = 6 per group. In (B) *p = 0.0347 and 0.0244, and in (D) **p = 0.0019 and 0.0016 by ANOVA followed by Tukey’s posthoc tests. Scale Bars: 50 μm. The data used to make this figure can be found in S1 Dataset.

To begin to determine possible mechanisms of the age-dependent vascular compromise, pericyte loss, and an increase in innate immune responses, transcript and protein expression levels of APOE were assessed in aging brains. APOE is a strong candidate to mediate age-related neurovascular decline for the following reasons. First, variations in human *APOE* are the major genetic risk factors for decreased longevity and AD [34,35,36]. Second, APOE has regulatory effects on cerebrovascular integrity and function through pericyte signaling [37,38]. Finally, APOE is an important mediator of innate immune responses [39,40,41]. Supporting a possible role for APOE in mediating age-related neurovascular dysfunction, *Apoe* transcript expression was noticeably decreased in the FPC (Fig 8A and 8B) and hippocampal CA1 (Fig 8C and 8D) in aged mice compared to young mice. Other studies have not reported an age-related decrease in APOE levels in the cortex and hippocampus [42]. Our data suggests this is because the decrease in *Apoe* transcript expression in the cortex was contrasted with a significant increase in *Apoe* transcript levels in the white matter regions, such as the CC in aged compared to young mice (Fig 8E–8G). This led to no measurable changes by RNA-seq (Fig 1), qPCR (4 mo versus 24 mo, 2^-ΔΔCt^ = 0.91) or western blotting (Fig 8H). Interestingly, *Apoe* was only expressed by astrocytes and not neurons or microglial cells (S8 Fig) and may reflect a difference between *Apoe* expression in normal aging compared to injury or disease where others have reported expression in additional cell types such as microglia [36]. The decline in astrocytic *Apoe* in the cortex was in contrast to a second astrocytic apolipoprotein, *ApoJ* (or Clusterin, *Clu*), where no significant changes were observed in aged compared to young brains (S9 Fig). Therefore, astrocytic *Apoe* expression is dramatically altered in localized regions of the brain during normal aging. This regional difference in *Apoe* expression could be due to differences between the type of astrocytes that populate the region (e.g., white matter astrocytes are mostly fibrous, while astrocytes in the cortex are mostly protoplasmic) or to different signals coming from the different environments [43,44]. Importantly, exercise preserved *Apoe* expression in the FPC (Fig 9A and 9B) and hippocampal CA1 (Fig 9C and 9D), adding further support for a role of APOE in age-related pericyte loss and neurovascular compromise.

**Fig 10:**
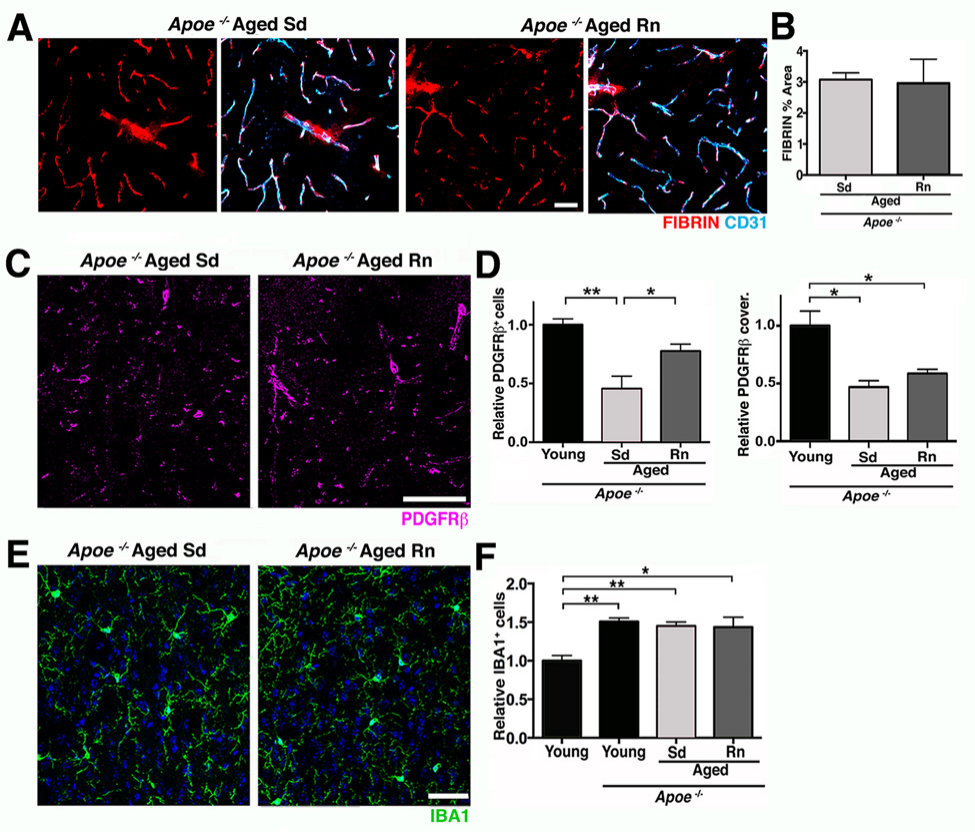
APOE is a contributor to exercise-induced preservation of neurovascular integrity. (A and B) Voluntary running does not change vascular and extravascular deposition of FIBRIN (red) in aged *Apoe* ^*-/-*^ mice. Endothelial cells (cyan) are immunolabeled with CD31. (C and D) PDGFRβ^+^ pericyte number (magenta) but not coverage, is only partially increased in aged Rn *Apoe* ^*-/-*^ mice compared to controls. (E and F) The number of IBA1^+^ microglia/monocytes (green) is not reduced by running in aged *Apoe* ^*-/-*^ mice. Values in (B, D, and F) are mean + SEM, (B) *n =* 6 and (E–G) *n =* 3–4 per group. In (D) **p = 0.0024, *p = 0.0468, *p = 0.0166, and *p = 0.0430, and in (F) **p = 0.0022 and 0.0084, and *p = 0.0105 by ANOVA followed by Tukey’s posthoc tests. Scale Bars: 50 μm. The data used to make this figure can be found in S1 Dataset.

To further investigate whether APOE contributes to neurovascular deterioration during normal aging and exercise-dependent preservation of the neurovascular unit, *Apoe*-deficient mice were exercised and compared to aged sedentary *Apoe*-deficient mice. Previous studies [37], confirmed by our study (S10 Fig), show neurovascular compromise including pericyte dysfunction or loss, vascular leakage, and BM reduction in young (<12 months old) *Apoe*-deficient mice that are strikingly similar to aged wild-type (*Apoe*-sufficient) mice. To determine the effect of exercise in the absence of APOE, *Apoe*-deficient mice were exercised from 12 months of age for 6 months, and components of the neurovascular unit assessed at 18 months of age. The average running distance performed by *Apoe*-deficient mice was not significantly different from aged *Apoe*-sufficient running mice (S10 Fig). However, age-related deficits in grip strength and nest construction were not prevented by exercised *Apoe*-deficient mice (S10 Fig). In the brain, exercise did not restore vascular leakage in *Apoe*-deficient mice (Fig 10C and 10E). Exercise only partially restored PDGFRβ^+^ pericyte number in aged *Apoe-*deficient mice (Fig 10D and 10F), but did not impact the density of COL4^+^ microvessels (S10 Fig). Furthermore, exercise did not prevent the increase in microglia/monocytes in *Apoe*-deficient mice that was seen in wild-type mice (Fig 10D–10G). Therefore, overall, exercise had little to no effect on age-related neurovascular dysfunction and microglia/monocyte numbers in *Apoe*-deficient mice.

## Discussion

**Fig 11:**
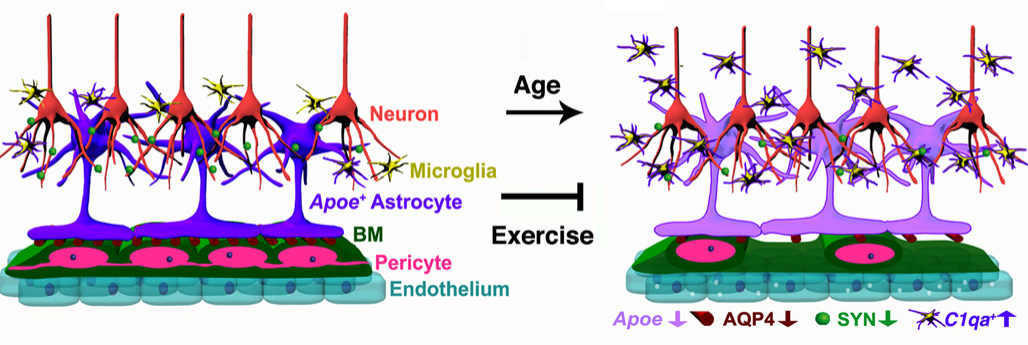
Schematic illustration of age-related changes in the neurovascular unit that are prevented by exercise. In the aged cortex of sedentary mice, neurovascular dysfunction is evident by decreased numbers of pericytes, decline in BM coverage, and increased transcytosis on endothelial cells. Expression of AQP4 in astrocyte endfeet and down-regulation of *Apoe* (purple) are also found as part of the age-related dysfunction of the neurovascular unit. In addition, decrease in synaptic proteins such as synaptophysin (SYN) is found in aged neurons. The number of proinflammatory IBA1^+^ microglia/monocytes expressing high levels of *C1qa* RNA is also increased in the aged cortex and HP indicating age-related neuroinflammation in aged mice. These age-related changes were successfully prevented by 6 months of voluntary running during aging, indicating the important contribution of physical activity on preservation of cerebrovascular function during aging.

Dysfunction of the neurovascular unit in the aging and aged brain is of great interest, since numerous studies have independently correlated the development of AD with vascular dysfunction during aging [2,6,45,46,47], but the mechanisms involved are not known. Studies have shown deterioration of the cerebrovascular ultrastructure along with decreasing CBF and lower metabolic rates of glucose and oxygen in normal human aging [45]. Similarly, exercise has been shown to be beneficial for the brain [4,9,11,12,13,48], but the processes positively impacted by exercise are not completely understood. Here, we elucidated the damaging effects of normal aging on the neurovascular unit in the cortex and HP of mice and show that exercise can prevent these detrimental changes (Fig 11).

Transcriptional profiling predicted dysfunction of the neurovascular unit particularly in the FPC, with some relevant genes also DE in the HP. Other studies have also profiled aging brains (e.g., [49,50,51]) but ours, to our knowledge, is the first to propose dysfunction of the neurovascular unit in aging mice. However, enrichment analyses of the DE genes comparing young and old male and female brains from the human study performed by Berchtold and colleagues (supplemental tables 3 and 4 from [51]) reveals overrepresentation of genes in multiple KEGG pathways relevant to the neurovascular unit (including focal adhesion, vascular smooth muscle, and ECM-receptor interactions) and neuroinflammation (including the complement cascade). These pathways are strikingly similar to the pathways we have identified in our study (Fig 1) providing compelling evidence that our findings in aging mice are directly relevant to human aging.

Genes relevant to the neurovascular unit were more represented in the transcriptional profiles from the FPC compared to the HP, adding further support to the possibility that specific brain regions may be more or less susceptible to aging than others [51]. However, some genes relevant to the neurovascular unit were DE in the HP, and neurovascular decline in CA1 region of the HP was observed. An explanation for region-specific differences in the transcriptional profiles may be due to the compositions of the tissues being profiled. For instance, there are significant differences in vascular density between the cortex and the HP [45,52]. In the rat brain, the highest vascular density is found in the neocortex (frontal and parietal regions profiled here) where variations in vascular density were shown to impact metabolic and synaptic activity [52]. This could result in increased sensitivity to detect neurovascular dysfunction by transcriptional profiling in the FPC compared to the HP. Also, the density and reactivity of astrocytes (compared to neurons) is different in the cortex compared to the HP with hippocampal astrocytes constitutively expressing GFAP, even in healthy brains [44,53]. This could also explain in part why, in our study, molecular pathways associated with neuroinflammation were more represented in the HP. Although others have also proposed region-specific changes in response to aging [51], further work is required to fully determine the extent of region-specific susceptibility in the aging brain.

Multiple changes to the neurovascular unit in the FPC and HP were observed in aged mice, one of the most striking being a significant loss of pericytes and their vascular coverage. While it is not known whether aging impacts pericytes directly or indirectly, pericyte function is critical for vessel stability, blood flow, and blood–brain barrier (BBB) integrity and function [19,54]. Loss of pericytes can cause BBB breakdown (by increasing endothelial transcytosis), reductions in BM, and alterations on AQP4 localization, correlating with an increase in innate immune responses [20,55,56], features observed in aged mice. Sporadic perivascular and more frequent intravascular deposits of fibrin, and increased vesicular transcytosis in endothelial cells in the aged cortex suggests vascular compromise is possibly occurring as a consequence of pericyte loss and/or dysfunction. Normal aging in humans is also associated with increased vascular compromise [57,58]. In fact, a recent study, using a new high resolution MRI method to map the blood-to-brain transfer constant of gadolinium (K_trans_) regionally and quantitatively, found that vascular leakage is an early event in the aged human brain that begins in the HP and is correlated with cognitive decline [58], supporting our findings in the aged mouse brain. Moreover, discontinuous and dysregulated coverage of BM on cortical microvessels and reduction of AQP4 protein levels at the neurovascular interphase were also observed in aging mice, suggesting that astrocyte–endothelial cell interactions are altered in the aged brain. It has been proposed that pericytes regulate the interactions of astrocytic endfeet with the vasculature [20], and age-related pericyte dysfunction and degeneration could lead to the disruptions of astrocyte-vascular interactions. Growing evidence supports age-related vascular dysfunction as a major contributing factor in the onset and progression of AD [6,7,45]. Extensive reductions in the number of pericytes have been correlated with BBB breakdown in postmortem AD brains [59], and intravascular and perivascular deposition of fibrin has been also found in AD mice [15,17] and AD patients [15,18,60]. Our data suggest that signaling changes between astrocytes, pericytes, and endothelial cells are impacted by aging, underpin neurovascular dysfunction, and may contribute to neurodegenerative diseases such as AD.

Our data also showed a direct correlation between pericyte loss, neurovascular decline, and an increase in *C1qa* (an initiating molecule of the classical pathway of the complement cascade) in response to aging. One key role of the neurovascular unit is to control the infiltration of peripheral immune cells (inflammation) and to protect the brain against blood-derived protein extravasation [45]. However, it is not clear whether the increase in *C1qa* ^+^ microglia/monocytes in aging mice is as a result of the proliferation of resident microglia or the infiltration of peripheral monocytes. During aging, a shift from an anti-inflammatory to a proinflammatory state can occur in the brain leading to cognitive decline, suggesting innate immune responses are an important characteristic of aging [8]. Conversely, an age-related increase of proinflammatory markers in the blood is correlated with poor cognitive performance in older adults [8], suggesting that age-related cognitive decline could be triggered in part by age-related systemic inflammation. Increased levels of *C1qa* have been identified in the brains of 12 mo mice [30], indicating that proinflammatory processes are detected early in aging brains. Moreover, age-related cognitive decline in mice is prevented by genetic deletion of *C1qa* supporting the deleterious effect of this protein and proinflammatory processes in brain function during aging [30]. Further work is required to determine whether peripheral complement-expressing monocytes infiltrate into the aging brain and whether the roles of complement-expressing resident microglia and/or peripheral monocytes are beneficial, damaging, or both. It is also not clear whether complement induction precedes neurovascular decline or occurs as a consequence of it. If the entry of peripheral immune cells into the brain is a key driver of age-related neurovascular decline, targeting peripheral immune cells before they enter the brain may be a feasible route to combat age-related cognitive decline and neurodegenerative diseases such as AD.

A major finding of this study is that exercise prevents neurovascular unit decline, including the reduction of age-related fibrin deposition and preservation of pericytes. The positive impact of exercise on neurogenesis, angiogenesis, neuronal activity, and cognition have been well demonstrated [4,9,11,13,61], but little is known about the effects of long-term exercise on cellular and molecular interactions of the neurovascular unit and other glial cells during aging. In our study, long-term exercise during aging prevented deficits in spontaneous and common behaviors in mice that are similar to ADL in humans, which are also affected by aging and improved by physical activity [62,63,64,65]. For instance, changes in grip strength and physical frailty in humans have been strongly associated with decline in cognitive performance [66,67,68]. It has been proposed that common biological processes mediate age-related decline in physical and cognitive function [67], suggesting that exercise in aging could be targeting and preventing declines in these common processes. Our data support this hypothesis by demonstrating that long-term exercise in aging mice prevents age-related physical frailty and behavioral deficits along with enhancements in brain structure and function.

While it is not yet known whether exercise-induced pericyte protection is direct or indirect, the importance of pericytes in the neurovascular unit underscores the potential of exercise to ameliorate age-associated degenerative changes and preserve long-term neurovascular health. Interestingly, previous reports found that levels of peripheral circulating platelet-derived growth factor subunit B (PDGFB), an essential growth factor for pericytes derived from endothelial cells [69], are significantly reduced with age [70], but increased by exercise [71]. The effects of peripheral growth factors on the cerebrovascular system have been demonstrated before. For instance, circulatory IGF1 is necessary for exercise-induced angiogenesis [72] in the young brain, while blood-derived GDF11 induces angiogenesis in the aged subventricular zone during heterochronic parabiosis [73], suggesting that circulatory factors could be mediating exercise-induced changes in the cerebrovascular system. Further studies are necessary to determine the possible contribution of circulatory factors to exercise-mediated improvements of cerebrovascular health.

Another important finding in this study was the significant reduction in the number of aged-induced proinflammatory *C1qa* ^+^ microglia/monocytes in exercised mice. Induction of *C1qa* in the CNS promotes synapse elimination and synaptic dysfunction during development, neurodegenerative diseases such as glaucoma and AD, and aging [29,30,31,32,74]. Importantly, the positive changes found in exercised mice were accompanied by stabilization of synaptic proteins and improvements in neuronal plasticity along with behavioral enhancements when compared with age-matched sedentary mice, and could be a direct result of a lessening of complement activation. Increased levels of C1QA are found in aged human brains suggesting a possible contribution of the complement cascade to age-related cognitive decline [30], but the impact of exercise on complement activation in the human brain has not been studied. However, it is well established that exercise in humans increases hippocampal volume and blood flow, induces greater levels of electrical and synaptic activity, and improves memory and cognitive performance in older adults that exercised [9,11,12]. This further highlights the importance of understanding the mechanisms by which aging and lifestyle directly or indirectly affect the function of astrocytes, pericytes, and microglia/monocyte.

Determining the mechanisms by which aging leads to neurovascular decline and how exercise prevents this decline is important, as it could lead to the identification of therapeutic strategies that target similar processes. Here we suggest APOE as a strong candidate for mediating age-related neurovascular unit decline; *Apoe* expression decreases in the cortex and HP of aged mice, *Apoe* expression is preserved by exercise, and exercise has little to no effect on behavioral deficits, neurovascular dysfunction, and innate immune responses in aged *Apoe*-deficient mice. Given that mice deficient in *Apoe* show vascular permeability, decreased CBF, synapse loss, and cognitive impairments [37,75,76], a decrease in *Apoe* expression in the aging brain would be predicted to impact the health of the neurovascular unit. Supporting this, a previous study shows an activation of a proinflammatory pathway in pericytes in *Apoe*-deficient mice leading to BM dysregulation and neurovascular breakdown [37]. Interestingly, although exercise had no effect on most components of the neurovascular unit in *Apoe*-deficient mice, exercise did partially increase pericyte number, but not coverage, indicating exercise has at least some positive effects on pericyte survival independent of APOE but they could be still dysfunctional. Another possibility is that intensity and duration of the exercise in this study was not sufficient for *Apoe*-deficient mice to reach the beneficial effects found in the wild-type mice. It is important to note that *Apoe*-deficient mice already had a dysfunctional neurovascular unit prior to running in contrast to the experiments using wild-type mice. Therefore, it is possible that exercise prevents neurovascular dysfunction but it has little to no effect on mice with an already dysfunctional neurovascular unit. This outcome would also be important, as neurovascular dysfunction from events such as brain injury or strokes could predispose to neurodegenerative diseases such as AD, and exercise may not be as beneficial in these circumstances.

Further work is needed to fully explore the role of *Apoe* in age-related neurovascular decline. In humans, *APOE* has three alleles that encode three different isoforms of the protein *APOE2*, *APOE3*, and *APOE4* that differ by a single amino acid substitution [77]. Human *APOE* alleles impact neurovascular function to different extents in mice with mice carrying human *APOE4* showing altered neurovascular function from a young age compared to mice carrying human *APOE2* or human *APOE3* [37]. Similar to *Apoe* deficiency, *APOE4* transgene in mice induces the activation of the proinflammatory cyclophilin A (CypA)–matrix metalloproteinase 9 (MMP-9) pathway in brain pericytes, leading to the breakdown of the BBB and neurodegeneration [37,38]. This proinflammatory pathway is also activated in the cerebrospinal fluid (CSF) of cognitively normal *APOE4* carriers [78] and in the microvessels of postmortem brains from AD patients [79]. Furthermore, increased pericyte degeneration and BBB breakdown are found in brains of *APOE4* carriers with AD supporting the role of *APOE4* in neurovascular dysfunction [79]. Interestingly, in humans and transgenic mice, *APOE4* carriers have lower levels of APOE protein in the brain compared to noncarriers [80,81], and human patients carrying *APOE4* develop AD 8–15 y earlier than carriers of *APOE2* or *APOE3* [38,62]. However, the APOE4 isoform also contributes to AD progression by binding with higher affinity to amyloid-β peptide and disrupting its clearance from the brain [82], and by contributing to amyloid-β aggregation [83]. Our findings suggest that deficiency or reduced efficiency of APOE in normal aging could cause dysfunction of neurovascular health and increased neuroinflammation, contributing to AD susceptibility, onset, and progression. Experiments to fully explore the role of APOE in age-related neurovascular decline and neuroinflammation, including conditional ablation of *Apoe* in aging mice and the impact of multiple human isoforms of *APOE*, are underway.

In summary, here we show that exercise in aging mice preserves the integrity and function of the neurovascular unit leading to a healthier and “younger-like” brain. In recent years, there has been a distinct lack of success in developing therapies that target specific components of AD such as plaque deposition and excessive Tau phosphorylation. Our data, supported by data from human studies [4,9,23,84], point towards focusing efforts on understanding the impact of aging and lifestyle on neurovascular unit decline and neuroinflammation, particularly pericyte dysfunction and loss, and activation of innate immune responses. Understanding these processes will both help encourage a healthy lifestyle that where possible includes exercise and could lead to the development of improved treatments for AD and other neurodegenerative disorders.

## Materials and Methods

### Mouse Strains

All experiments involving mice were conducted in accordance to policies and procedures described in the Guide for the Care and Use of Laboratory Animals of the National Institutes of Health and were approved by the Animal Care and Use Committee at The Jackson laboratory. All mice were bred and housed in a 12/12-hour light/dark cycle. As part of the Nathan Shock Center of Excellence in the Basic Biology of Aging at The Jackson Laboratory, cohorts of C57BL/6J mice were aged to between 12 and 24 months of age. All mice used in this study were females C57BL/6J (B6) (Stock number 00664). Female B6 mice homozygous for the *Apoe* ^*tm1Unc*^ mutation (*Apoe* ^*-/-*^) were obtained from The Jackson Laboratory (Stock Number 002052). To produce experimental animals, *Apoe* ^*-/-*^ mice were intercrossed and aged. For this study, mice under 9 months old were classified as “young” mice, while 12-month-old mice were classified as “aging” mice, and 18–24 month old mice were identified as “aged” mice. For the parameters analyzed in this paper, no significant differences were found between 18- and 24-month-old mice.

### Mouse Perfusion and Tissue Preparation

Mice were anesthetized with a lethal dose of ketamine/xylazine and transcardially perfused with 1X PBS to remove any trace of blood from the brain. After perfusion, mice were decapitated with brains carefully dissected out and hemisected in the midsagittal plane. For RNA-seq, the superior region of the cortex (region 1), the HP (region 2), and the RB (region 3) were removed and immediately snap-frozen. For other histological procedures, one-half of the hemisected brain was snap-frozen and the other half was fixed by immersion in 4% paraformaldehyde for two nights. After fixation, brains were rinsed in 1X PBS, immersed in 30% sucrose/PBS solution overnight at 4°C, frozen in OCT, and cryosectioned at 20 μm.

### RNA Extraction, Library Construction, and Sequencing

Tissues were homogenized with TRIzol reagent (Life Technologies) and centrifuged to remove debris. Chloroform (0.2 ml per 1 ml of TRIzol) was added to the cleared homogenate for phase separation. Total RNA was purified from the aqueous layer using the QIAGEN miRNeasy mini extraction kit (QIAGEN) according to the manufacturer’s instructions. RNA quality was assessed with the Bioanalyzer 2100 (Agilent Technologies). Poly(A) selected RNA-seq sequencing libraries were generated using the TruSeq RNA Sample preparation kit v2 (Illumina) and quantified using qPCR (Kapa Biosystems). Using Truseq V4 SBS chemistry, all libraries were processed for 125 bp paired-end sequencing on the Illumina HiSeq 2,500 platform according to manufacturer’s instructions.

### RNA-seq Analysis, Official Gene Names, and Gene Set Enrichment

Samples were subjected to quality control analysis by NGSQCtoolkit v 2.3 [85]. Reads with 70% of their bases having a base quality score ≥ 30 were retained for further analysis. Read alignment and expression estimation was performed using RSEM v 1.2.12 [86] with supplied annotations at default parameters against the C57BL/6J mouse genome (build-mm10). Bamtools v 1.0.2 [87] were used to calculate the mapping statistics. Differential gene expression analysis between groups was performed using EdgeR v 3.8.5 [87,88,89] following the removal of outlier samples and lowly expressed genes (cpm [or counts per million] < 1 in less than two samples). Normalization was performed using the trimmed mean of M values (TMM). Adjustment for multiple testing was performed using FDR. Genes were considered to be significantly DE at a FDR < 0.05. All raw and processed data is being made available through GEO archives and S1–S3 Tables. Official ENSEMBL gene IDs for all genes included in this study are provided in column 1 of S1, S2 and S3 Tables.

The Database for Annotation, Visualization and Integrated Discovery (DAVID, [89]) was used to add functional annotation to DE gene lists and provide statistical assessment of the annotations. We focused on the KEGG pathways [90]. For each list of DE genes, DAVID determines the number of genes in each KEGG pathway and uses a Fisher exact test to determine the probability that the number of genes in each pathway would have occurred by chance [89]. A *p*-value < 0.05 was used to identify significant pathways. Pathways with gene expression changes represented by colors (red–up-regulated, green–down-regulated) were generated at the KEGG website [90].

### Immunostaining

For immunostaining involving antibodies for vascular associated proteins, sections were pretreated with Pepsin as previously described [91] with minor modifications. Sections were hydrated with H_2_O for 3 min at 37°C followed by treatment of the tissue with 0.5 mg/ml of Pepsin (Sigma-Aldrich) for 18 min at 37°C. Sections were then rinsed twice with 1X PBS at room temperature (RT) for 10 min. After Pepsin pretreatment, sections were rinsed once in 1X PBT (PBS + 1% Triton 100X) and incubated in primary antibodies diluted with 1X PBT + 10% normal goat or normal donkey serum over two nights at 4°C. After incubation with primary antibodies, sections were rinsed three times with 1X PBT for 10 min and incubated for two hours in the corresponding secondary antibodies (1:800, Invitrogen). Tissue was then washed three times with 1X PBT for 10–15 min, incubated with DAPI and mounted in Poly aquamount (Polysciences). The following primary antibodies were used: goat anti-COL4 (1:40, R&D), goat anti-PDGFRβ (1:40, R&D), goat anti-CD31 (1:40, R&D), rabbit anti-LAM (1:200, Sigma-Aldrich), goat anti-mouse APOE (1:50, Santa Cruz Biotech), Biotinylated Lycopersicon Esculentum (Tomato) Lectin (1:200, Vector), rabbit anti-IBA1 (1:200, Wako), rabbit anti-GFAP (1:200, Dako), rabbit anti-AQP4 (1:200, Sigma-Aldrich), mouse anti-synaptophysin (SYN, 1:200, Millipore), mouse anti-NeuN (1:300, Millipore), rabbit anti-fibrinogen (FIBRIN, 1:200, DAKO). Blocking serum was not included in primary antibody solutions that contained the fibrinogen antibody.

For quantitative analysis of FIBRIN and SYN in the cortex, four images were randomly taken in the parietal cortex for each brain for each mouse and opened in ImageJ (1.47 d) software as a black and white image as reported previously [92]. Stained intensity and % of area statistics were obtained by generating surface segmentation using the same threshold criteria for all the pictures. For quantification of PDGFRβ^+^ or IBA1^+^ cells in the cortex and CA1, four images were taken for each brain from each mouse with a Zeiss Axio Imager fluorescent microscope and manually counted using the cell counter plugin from the ImageJ (1.47 d) software. For quantification of pericyte coverage of microvessels, the length PDGFRβ^+^ pericytes were calculated using the NeuronJ plugin in ImageJ (1.47d) software. All image analyses were performed blind to the experimental conditions.

### Vascular Imaging Analysis

For quantification of COL4^+^ and CD31^+^ capillary area in the cortex and CA1, six images were taken for each brain from each mouse with a Zeiss Axio Imager fluorescent microscope. Vessel area was quantified using an automated method developed in-house, available upon request. Briefly, a segmentation algorithm, modified from the ImageJ plugin VNT (Vascular Network Toolkit, http://ntwrkanlystlkit.sourceforge.net/), was used for measure blood vessels area on 20x magnification images that were stained with the vascular markers COL4, LAM, CD31, and Lectin. This segmentation algorithm was written as an ImageJ macro for automated processing of images that includes the following steps: Gaussian blur (2 px), “Find edges”, Variance (5 px), Median (3 px), Subtract (σ px), Multiply (255), Invert and Analyze Particles (S11 Fig). Image analysis was automated and blind to the experimenters. This automated processing had a positive correlation with quantification of vessel area by manual tracing (in ImageJ) performed by two independent investigators (R^2^ = 0.612, *p* = 1.24e^-07^) (S11 Fig).

### In Situ Hybridization and Analysis

For in situ hybridization experiments, RNA probes for mouse *Apoe* (GE Dharmacon Clone ID 5136415), *Arc* (GE Dharmacon Clone ID 3498057), *Clu* (GE Dharmacon Clone ID 30550773), and *C1qa* (GE Dharmacon Clone ID 3592169) were synthesized, labeled with digoxigenin (Dig), and hydrolized by standard procedures. Frozen sections were postfixed (4% PFA for 5 min), rinsed twice with 1X PBS, and acetylated with 0.25% acetic anhydride for 10 min in 0.1 M triethanolamine (TEA). Sections were then washed in PBS and incubated overnight at 65°C with hybridization solution (50% formamide, 1X Hybe solution [Sigma-Aldrich], 1 mg/ml yeast RNA) containing 1 μg/ml Dig-labeled riboprobe. After hybridization, sections were washed by immersion in 0.2XSSC (Sodium Chloride- Sodium Citrate) at 72°C for 1 hr. Dig-labeled probes were detected with an AP-conjugated anti-Dig antibody (Roche) followed by NBT/BCIP (nitroblue tetrazolium/5-bromo-4-chloro-3-indolyl phosphate) reaction (Roche). After in situ hybridization, sections were incubated in primary antibodies (GFAP, NEUN and IBA1) as previously described [29] and briefly described above. Sections were then incubated with DAPI for nuclei staining and mounted in Aqua PolyMount (Polysciences).

For quantitative analysis of *Apoe* and *Clu* RNA expression, the % area stained by the riboprobes in the cortex and CA1 was measured with ImageJ (1.47 d) software as reported previously [92]. Briefly, six images were taken in the parietal cortex for each brain from each mouse and opened in Image J as a black and white image. All images were converted to one stack and cropped to obtain just the center of the picture. Stained area statistics were obtained by generating surface segmentation using identical threshold criteria for all the pictures. All image analyses were performed blind to the experimental conditions. For quantification of *Arc* ^*+*^ or *C1qa* ^+^ cells in the cortex, four images were taken from each brain for each mouse in the transmission light channel and the fluorescence channel for fluorescently-labeled NEUN or IBA1 and manually counted using the cell counter plugin from the ImageJ (1.47 d) software.

### Transmission Electron Microscopy (TEM)

Mice were perfused with a mix solution of 2% PFA and 2% glutaraldehyde in 0.1 M Cacodylate buffer (Caco). After perfusion, brains were fixed overnight in the same solution at 4°C. The samples were then rinsed three times in 0.1 M Caco buffer. 100 μm thick coronal sections were cut on a vibrating microtome and post fixed with 2% osmium tetroxide in 0.1 M Caco buffer for 2 h at room temperature. Sections were rinsed three times with Caco buffer and then dehydrated through of series of alcohol gradations. The sections were then put into a 1:1 solution of propylene oxide/Epon Araldite (Electron Microscopy Sciences, Hatfield, PA) overnight on an orbital rotator. Sections were then flat embedded with 100% Epon Araldite between 2 sheets of Aclar film and polymerized at 65°C for 48 h. Specific areas of the parietal cortex in these sections were then selected, cut out with a razor blade and glued onto dummy blocks of Epon Araldite. 90 nm ultrathin sections were cut on a Diatome diamond knife, collected on 300 mesh copper grids, and stained with Uranyl Acetate and lead citrate. Grids were viewed on a JEOL JEM1230 transmission electron microscope and images collected with an AMT high-resolution digital camera. Twenty cross-sectional blood vessels were imaged per brain/mouse, *n* = 3 per group young (4 mo) and aged (18 mo) group.

### RNA and Protein Extraction with TRIzol

Hemisected brains were dissected as described above and the superior region of the cortex containing the parietal cortex was sliced, snap frozen at the time of collection and stored at −80°C. RNA extraction was performed according to the TRIzol (Invitrogen) manufacturer’s instructions. Briefly, tissue was homogenized in 1ml of TRIzol reagent per 50–100 mg of tissue sample, followed by separation of phases using chloroform and removal of the aqueous phase for RNA precipitation. The interphase and organic phenol-chloroform phase were saved for protein extraction. For protein isolation, 100% ethanol was added to the interphase/phenol-chloroform phase, centrifuged and the phenol-ethanol supernatant was taken. Protein was precipitated from the saved supernatant with isopropanol, washed with 0.3 M guanidine hydrochloride in 95% ethanol and resuspended in a 1:1 solution of 8 M urea (in Tris-HCl 1 M, pH 8.0) and 1% SDS using sonication as described previously [93]. Isolated RNA and protein were stored at −80°C until use.

### Quantitative PCR

Trizol-extracted RNA was used to assess the expression levels of mouse *Apoe* in young (*n* = 4) and aged (*n* = 4) cortex, with all samples normalized to β*-Actin*. RNA was treated with DNAse and reverse transcribed using the GeneAmp RNA PCR kit (Applied Biosystems). For quantitative PCR, the Quanti-Fast SYBR Green kit (Qiagen) was used and reactions were carried out with the following primers: *Apoe* (Forward: 5’-GGGCAAACCTGATGGAGAAG-3’ and Reverse: 5’-CCTGGCTGGATATGGATGTTG-3’)*;* and *β-Actin* (Forward: 5’-TGGAATCCTGTGGCATCCATGAAAC-3’ and Reverse: 5’-TAAAACGCAGCTCAGTAACAGTCCG-3’), with each gene being interrogated in triplicate. Ct (threshold cycle) was calculated as the mean of the successful replicates for each gene. For normalization, ΔCt values were calculated as Ct (gene of interest) minus geometric mean of Ct for the normalizers. The average and standard deviation of the young cortex were then calculated. The ΔΔCt was defined as the ΔCt (gene of interest) minus ΔCt (control mean). The fold change was calculated as 2 minus ΔΔCt (up-regulated) or –2ΔΔCt (down-regulated). A gene was considered DE if the fold change was greater than two standard deviations away from the mean fold change from the young cohort.

### Western Blot Analysis

Protein samples were separated by SDS-PAGE gel electrophoresis and transferred to nitrocellulose membrane. Before incubation with primary antibodies, membranes were blocked in 5% milk, and after primary antibody incubation the appropriate peroxidase-conjugated antibody (Millipore) was used as a secondary antibody. For detection, membranes were treated with the Amersham ECL western blotting analysis system (GE Healthcare) and exposed to the High performance chemiluminescence film (GE Healthcare). The primary antibodies used for immunoblotting are: goat anti-mouse APOE (1:1,000, Millipore), rabbit anti-pan laminin (LAM, 1:1,000, Sigma-Aldrich), mouse anti-PSD95 (1:1,000, Millipore), and mouse anti-βActin (1:2,000, Abcam).

### Behavioral Tests

Fore limb strength was assessed by the suspended grid-grasping test. Mice were timed for how long they can support their body weight by holding onto a metal mesh suspended in midair. One minute was established as the maximum time for the test. Nest construction was assessed as reported previously [94,95]. Briefly, singly housed mice were provided with a preweighted nestlet one hour before the dark cycle. The next morning, the nest construction was assessed following the 1 to 5 scoring method established by the Deacon lab (2012). To evaluate burrowing, individually caged mice were provided with a one open ended 200 mm long and 70 mm diameter polyvinyl chloride (PVC) plastic tube filled with 200 g of mouse food pellets as described previously [94]. The open end was elevated 3 cm off the bottom of the cage with machine screws (5 cm long). The mice were allowed to burrow for 2 hr, and the amount of food pellets that remained in the tube was calculated. All behavioral tasks were performed at least three times and the average calculated.

### Exercise by Voluntary Running

For voluntary wheel running, mice were given free access to running saucer wheels (Innovive Inc) (day and night, two mice per cage). Sedentary mice had no access to wheels. Both groups were housed under these conditions for 6 mo. Animals were tested for running capacity by placing individual mice in a cage with a wireless saucer wheel (ENV-044 Med Associates Inc.) for 10 d. Data were collected nightly (16 hr), analyzed and average distance ran per night for each mouse calculated.

### Statistical Analysis

Data were analyzed using GraphPad Prism software. Significance was calculated using unpaired *t* tests for comparisons between two groups and one-way multifactorial analysis variance (ANOVA) followed by Tukey posthoc tests for multiple comparisons. *p*-values are provided as stated by GraphPad Prism software and significance was determined with *p*-values less than 0.05.

## Supporting Information

S1 Dataset Original data for each of the figures in the manuscript and supporting information. (XLSX)

S1 Fig Decrease in BM coverage with age. (A) COL4^+^ microvessels are significantly decreased in the cortex of aged mice when compared with young B6 mice. (B) Quantification of COL4^+^ and CD31 capillary area in the young and aged B6 cortex. (C) Colocalization of COL4 (green) with LAM (magenta) and Lectin (blue) in young and aged B6 mice. Loss of COL4 and LAM coverage in Lectin^+^ capillaries is evident in the aged mouse when compared with the young. In (B), values are relative mean + SEM to the young values, *n* = 4–6 mice per group. **p = 0.005 by unpaired *t* test. Scale Bars: 50 μm. The data used to make this figure can be found in S1 Dataset.
(TIF)

S2 Fig Irregularities in BM coverage with age. (A) COL4 and LAM immunostaining in the cortex of young and aged mice showing irregularities in the coverage of microvessels by these BM proteins. Arrows indicates loss of coverage while arrowheads show increased levels deposition of these proteins in microvessels. (B) Total levels of LAM protein in the cortex of young and aged mice. In panel (B), values are mean + SEM, *n* = 4 mice per group. Scale Bars: 50 μm. The data used to make this figure can be found in S1 Dataset.
(TIF)

S3 Fig BM and pericyte coverage are decreased with age in the CA1 region of the HP. (A) COL4^+^ microvessels are significantly decreased in the cortex of aged mice when compared with young B6 mice. (B) Microvessels coverage of PDGFRβ+pericytes is significantly reduced in the hippocampal CA1 region. In (A) and (B), values are relative mean + SEM to the young values, *n* = 6 mice per group. ***p = 0.0001 and ****p < 0.0001 by unpaired *t* test. Scale Bars: 50 μm. The data used to make this figure can be found in S1 Dataset.
(TIF)

S4 Fig Synaptic proteins are decreased with age but preserved by exercise. (A) SYN cortical immunoreactivity is noticeably decreased in aged (24 mo) mice when compared with young (4 mo) mice. (B) Levels of intensity of SYN immunoreactivity were significantly decreased in aged mice when compared with young and aging mice. (C) PSD-95 protein levels in the cortex are significantly decreased in aged mice when compared with young mice. (D–E) Voluntary running significantly preserves SYN cortical immunoreactivity in aged mice. In panels (C and D), values are relative mean + SEM to the young values (*n* = 4). In (B) ***p = 0.0003, and in (E) **p < 0.0001 by ANOVA followed by Tukey’s posthoc tests and in (C) *p* < 0.005 by unpaired *t* test. Scale Bars: 50 μm. The data used to make this figure can be found in S1 Dataset.
(TIF)

S5 Fig BM dysregulation in aged brains is prevented by exercise. (A) COL4^+^ microvessels are significantly increased in the cortex of aged runner mice when compared with aged sedentary mice. (B) Quantification of CD31^+^ capillary area shows no significant differences between groups. (C) COL4^+^ microvessels are significantly increased in the CA1of aged runner mice when compared with aged sedentary mice. (D) Quantification of CD31^+^ capillary area in the CA1 shows no significant differences between groups. In (B–D), values are relative mean + SEM to the young values, *n* = 4 aging mice, *n* = 6 aged sedentary mice and *n* = 6 aged runner mice. In (A) ***p = 0.0007 and in (C) *p = 0.0163 by ANOVA followed by Tukey’s posthoc tests. Scale Bars: 50 μm. The data used to make this figure can be found in S1 Dataset.
(TIF)

S6 Fig Astrocyte reactivity is attenuated in aged, exercised mice. (A) Increased GFAP immunoreactivity in the cortex of aged sedentary mice is prevented by voluntary running in aged runner mice. (B) Quantification of GFAP^+^ area in the cortex of aging, aged sedentary and aged runner mice. Values are mean + SEM of the % area immunolabeled with GFAP, *n* = 4 mice per group. *p < 0.05 by ANOVA followed by Tukey’s posthoc tests. Scale Bar: 50 μm. The data used to make this figure can be found in S1 Dataset.
(TIF)

S7 Fig Decline in pericyte and AQP4 coverage in the CA1 region of aged mouse brains are prevented by exercise. (A–B) PDGFRβ^+^ pericyte coverage is significantly increased in the CA1 region of aged runner mice when compared with aged sedentary mice. No changes in the number of PDGFRβ^+^ pericyte were found between groups. (C–D) AQP4^+^ microvessels are significantly increased in the CA1of aged runner mice when compared with aged sedentary mice. In (B and D), values are relative mean + SEM to the young values, *n* = 4 aging mice, *n* = 6 aged sedentary mice, and *n* = 6 aged runner mice. In (B) **p = 0.0011 and ***p = 0.0010 and in (D) ***p = 0.0009 and *p = 0.0183 by ANOVA followed by Tukey’s posthoc tests. Scale Bars: 50 μm. The data used to make this figure can be found in S1 Dataset.
(TIF)

S8 Fig *Apoe* expression is only observed in astrocytes in the cortex of the aging mouse brain. (A) *Apoe* in situ hybridization signal (purple/white) colocalized with GFAP immunostaining (green) in astrocytes. (B) *Apoe* expression (purple/white) is absent on IBA1^+^ microglia and NEUN^+^ neurons. Scale Bars: 50 μm.
(TIF)

S9 Fig Clusterin (*Clu*) is expressed mainly in astrocytes and, in contrast to Apoe, does not decline with age. (A) *Clu* in situ hybridization signal (purple/white) colocalized with GFAP (green/astrocytes), IBA1 (green/microglia) and NEUN (red/neurons) immunostaining. Arrows indicate colocalization of GFAP with *Clu* expressing cells, but not IBA1^+^ microglial cells. Arrowheads show cells positive for *Clu* signal are negative for NEUN immunostaining. (B–C) *Clu* expression (purple) does not change with age in the neocortex. (D–E) No changes in *Clu* expression are found in the CC. In (C and E), values are relative mean + SEM to the young values, *n* = 4 mice per group. Scale Bars: 50 μm (A and D), 100 μm (B). The data used to make this figure can be found in S1 Dataset.
(TIF)

S10 Fig Deficiency of *Apoe* promotes early neurovascular decline. (A) Col4^+^ capillaries are significantly reduced on APOE-deficient mice. In (A) lower panel, merge images showing PDGFRβ^+^ pericytes (magenta), IBA1^+^ microglia (green) and LECTIN^+^ endothelial cells in the young *Apoe* ^*+/+*^ and *Apoe* ^*-/-*^ mice. (B) Quantification of COL4^+^ capillary area between young (9 mo) *Apoe* ^*+/+*^ and *Apoe* ^*-/-*^ B6 mice. (C) Quantification of CD31^+^ capillary area, nonsignificant changes are observed. (D) Quantification of PDGFRβ^+^ pericytes showing a significant decrease of these cells in the APOE-deficient mice. (E) Quantification of IBA1^+^ microglia showing a significant increase of these cells in the APOE-deficient mice. (F) Running distances by *Apoe* ^*-/—*^aged mice were not statistically significant from *Apoe* ^*+/+*^-aged runner mice. (G) Deficits in grip strength found in *Apoe* ^*-/-*^ aging and aged sedentary mice were not statistically significant from *Apoe* ^*-/-*^ aged runner mice, although a partial increase is observed. (H) Deficits in nest construction behavior were not preserved in *Apoe* ^*-/—*^aged running mice when compared with *Apoe* ^*-/—*^aged sedentary mice. (I–J) No changes in COL4^+^ capillary area were found between aged sedentary *Apoe* ^*-/-*^ and aged runner *Apoe* ^*-/-*^ mice. (K) Quantification of CD31^+^ capillary area in young *Apoe* ^*-/-*^, aged sedentary *Apoe* ^*-/-*^ and aged runner *Apoe* ^*-/-*^ mice. In panels (B, D, E, F–H, J, and K) values are mean + SEM. In (B, D, and E) **p < 0.005, *n* = 4 by unpaired *t* test. In (H) *p = 0.0416 and ***p = 0.0005 by ANOVA followed by Tukey’s posthoc tests. Scale Bars: 50 μm. The data used to make this figure can be found in S1 Dataset.
(TIF)

S11 Fig Automated calculation of vascular area in the mouse cortex. (A–C) Sample images showing steps of image-automated processing for microvessels area quantification. Original image show in A, invert step after segmentation show in B and analyzed structures in C. Inset on C shows the tabulated data acquired after the analysis. (D) A positive correlation (R^2^ = 0.612, *p* = 1.24e^-07^) is observed between vascular area calculation by manual segmentation versus automated segmentation by the segmentation algorithm developed in our lab.
(TIF)

S1 Table DE genes in the FPC/CC (region 1). (XLSX)

S2 Table DE genes in the HC (region 2). (XLSX)

S3 Table DE genes in the RB (region 3). (XLSX)

S4 Table KEGG Pathway Analysis (All regions). (XLSX)

## Abbreviations

AD: Alzheimer disease
ADL: activities of daily living
AQP4: aquaporin-4
*Arc*: activity-regulated cytoskeletal gene
B6: C57BL/6J
BBB: blood–brain barrier
BM: basement membrane
CBF: cerebral blood flow
CC: corpus callosum
CSF: cerebrospinal fluid
COL4: collagen IV
CypA: cyclophilin A
DE: differentially expressed
ECM: extracellular matrix
FPC: frontoparietal cortex
GFAP: glial fibrillary acidic protein
HP: hippocampus
KEGG: Kyoto Encyclopedia of genes and genomes
LAM: laminin
MMP-9: matrix metalloproteinase 9
PDGFB: Platelet-derived growth factor subunit B
RB: rest of the cortex and brain stem
SEM: Standard Error of the Mean
