# Supplementary figures and images for: APOE Stabilization by Exercise Prevents Aging Neurovascular Dysfunction and Complement Induction
Source: PLoS Biol. 2015 Oct 29;13(10):e1002279. doi: 10.1371/journal.pbio.1002279 (PMC4626092; doi:10.1371/journal.pbio.1002279)

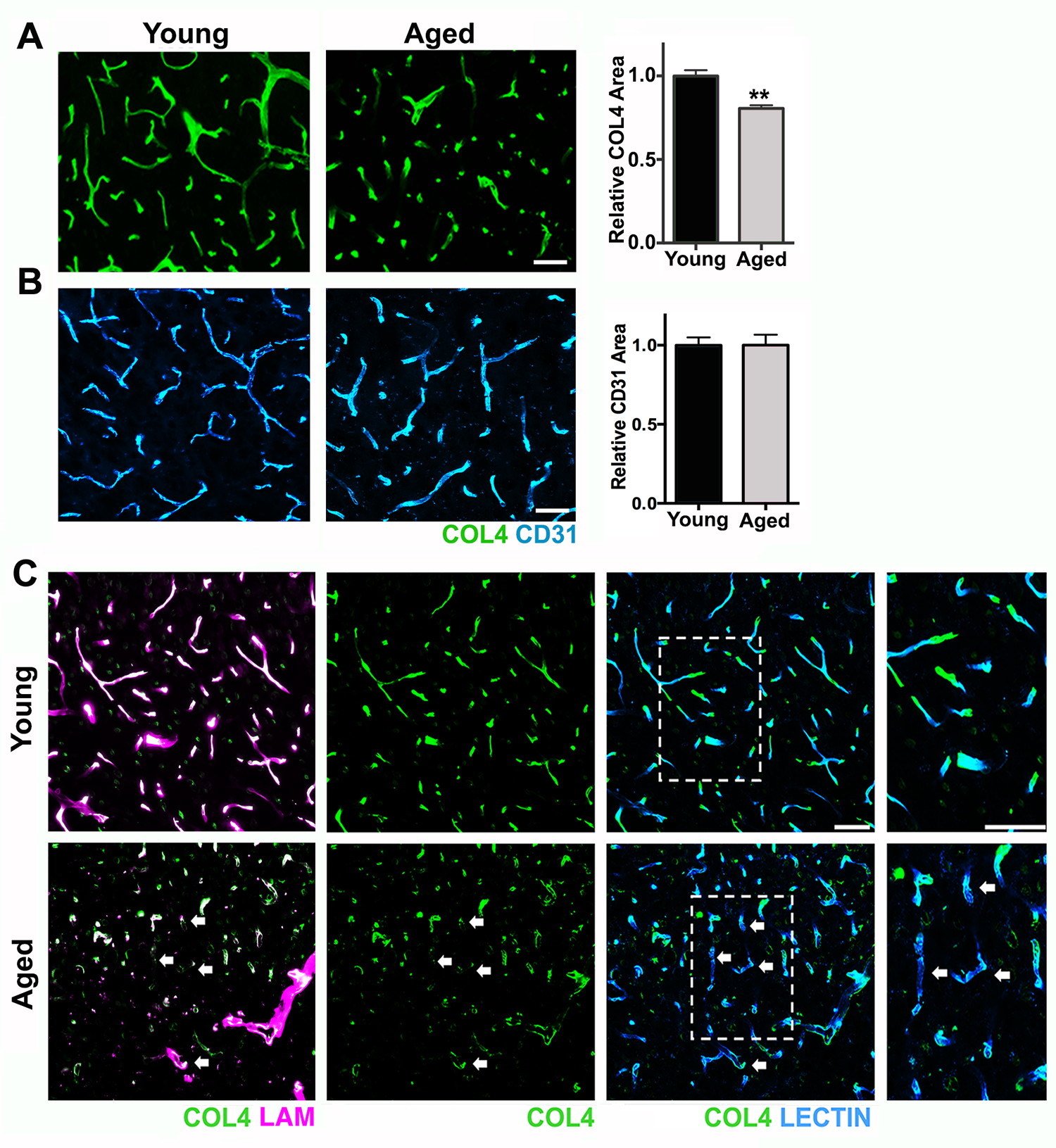

Supplement: S1 Fig — (A) COL4+ microvessels are significantly decreased in the cortex of aged mice when compared with young B6 mice. (B) Quantification of COL4+ and CD31 capillary area in the young and aged B6 cortex. (C) Colocalization of COL4 (green) with LAM (magenta) and Lectin (blue) in young and aged B6 mice. Loss of COL4 and LAM coverage in Lectin+ capillaries is evident in the aged mouse when compared with the young. In (B), values are relative mean + SEM to the young values, n = 4–6 mice per group. **p = 0.005 by unpaired t test. Scale Bars: 50 μm. The data used to make this figure can be found in S1 Dataset. (TIF) [file pbio.1002279.s002.tif]

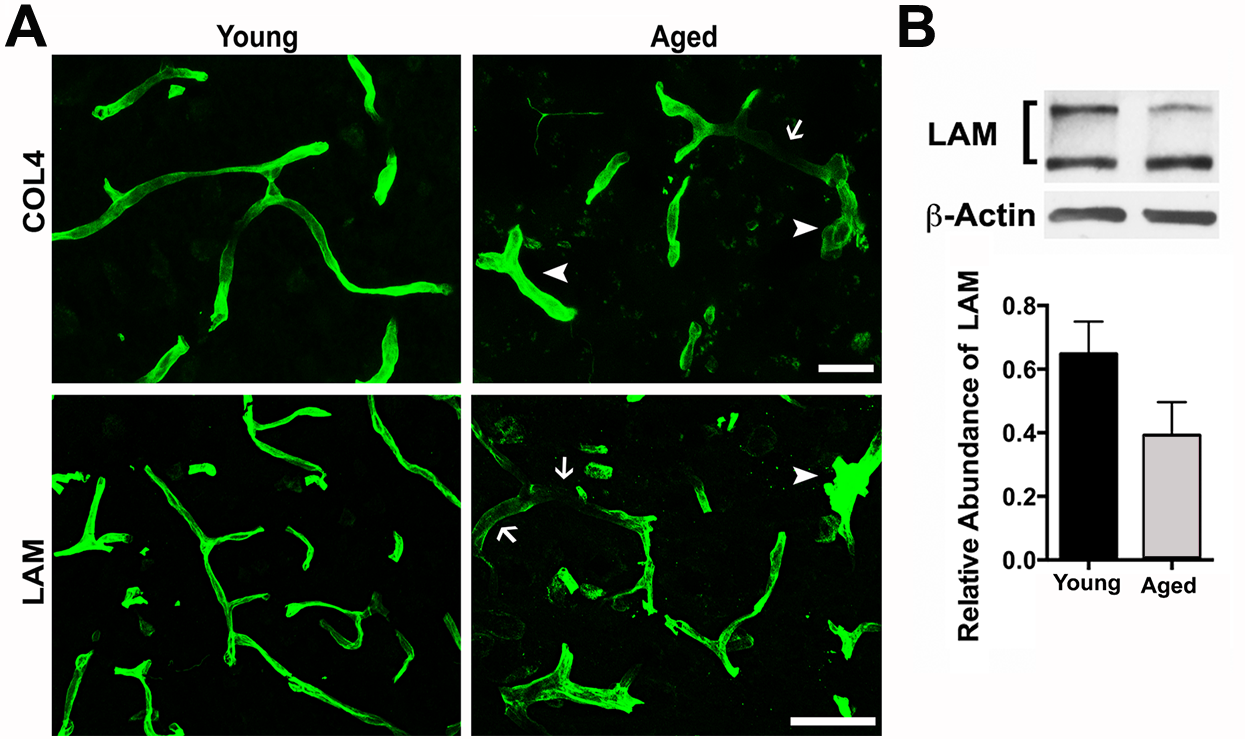

Supplement: S2 Fig — (A) COL4 and LAM immunostaining in the cortex of young and aged mice showing irregularities in the coverage of microvessels by these BM proteins. Arrows indicates loss of coverage while arrowheads show increased levels deposition of these proteins in microvessels. (B) Total levels of LAM protein in the cortex of young and aged mice. In panel (B), values are mean + SEM, n = 4 mice per group. Scale Bars: 50 μm. The data used to make this figure can be found in S1 Dataset. (TIF) [file pbio.1002279.s003.tif]

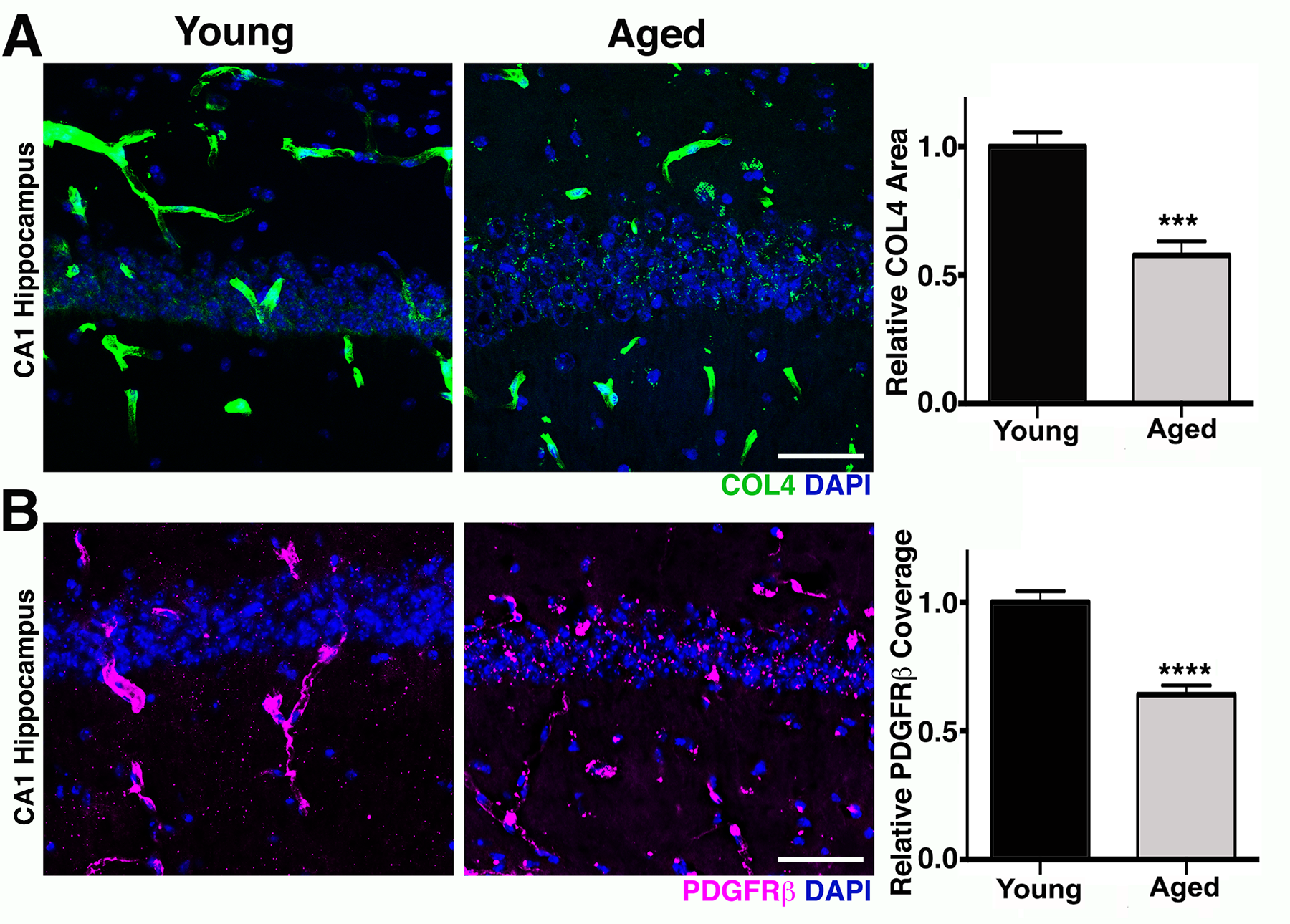

Supplement: S3 Fig — (A) COL4+ microvessels are significantly decreased in the cortex of aged mice when compared with young B6 mice. (B) Microvessels coverage of PDGFRβ+pericytes is significantly reduced in the hippocampal CA1 region. In (A) and (B), values are relative mean + SEM to the young values, n = 6 mice per group. ***p = 0.0001 and ****p < 0.0001 by unpaired t test. Scale Bars: 50 μm. The data used to make this figure can be found in S1 Dataset. (TIF) [file pbio.1002279.s004.tif]

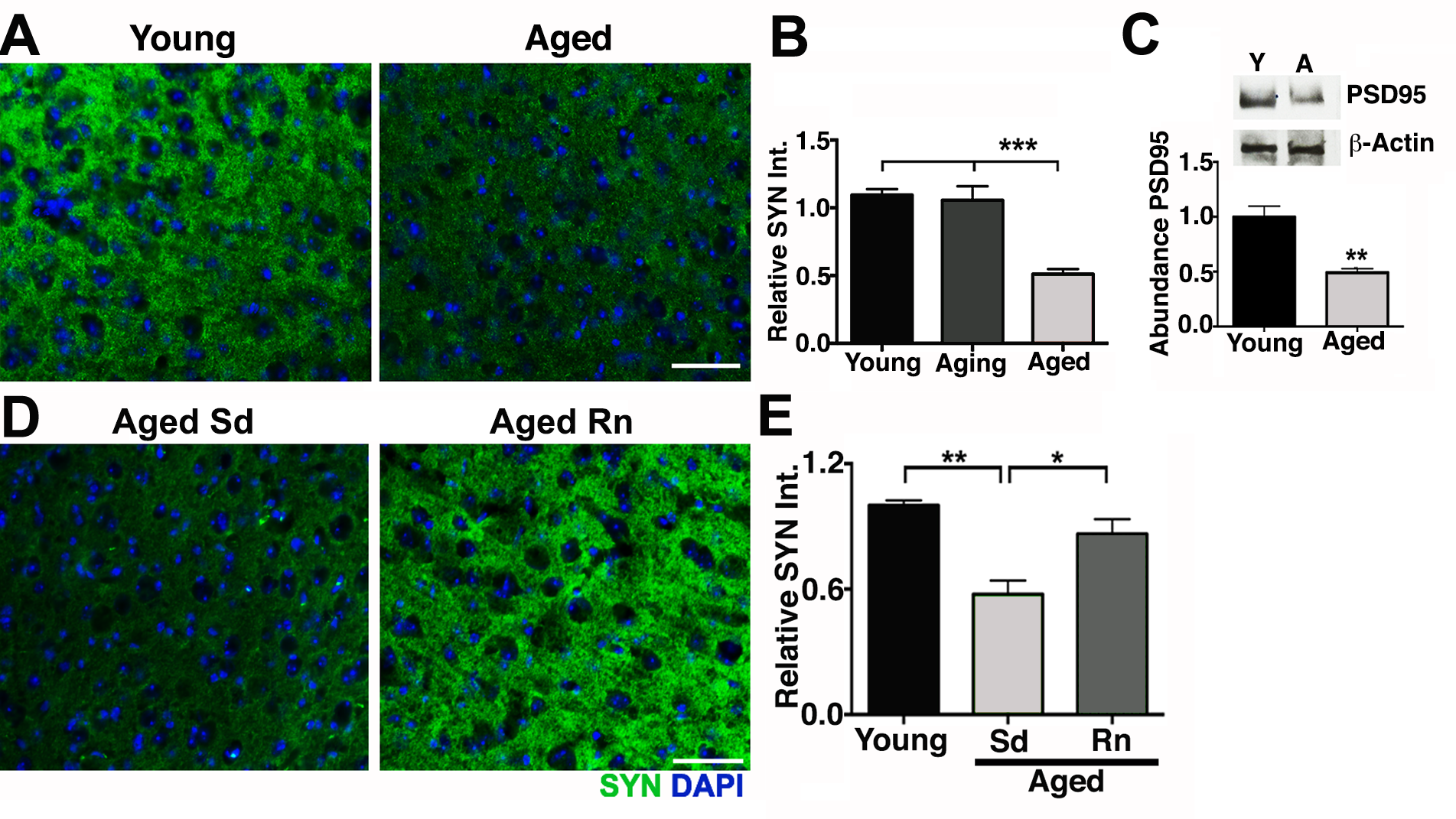

Supplement: S4 Fig — (A) SYN cortical immunoreactivity is noticeably decreased in aged (24 mo) mice when compared with young (4 mo) mice. (B) Levels of intensity of SYN immunoreactivity were significantly decreased in aged mice when compared with young and aging mice. (C) PSD-95 protein levels in the cortex are significantly decreased in aged mice when compared with young mice. (D–E) Voluntary running significantly preserves SYN cortical immunoreactivity in aged mice. In panels (C and D), values are relative mean + SEM to the young values (n = 4). In (B) ***p = 0.0003, and in (E) **p < 0.0001 by ANOVA followed by Tukey’s posthoc tests and in (C) p < 0.005 by unpaired t test. Scale Bars: 50 μm. The data used to make this figure can be found in S1 Dataset. (TIF) [file pbio.1002279.s005.tif]

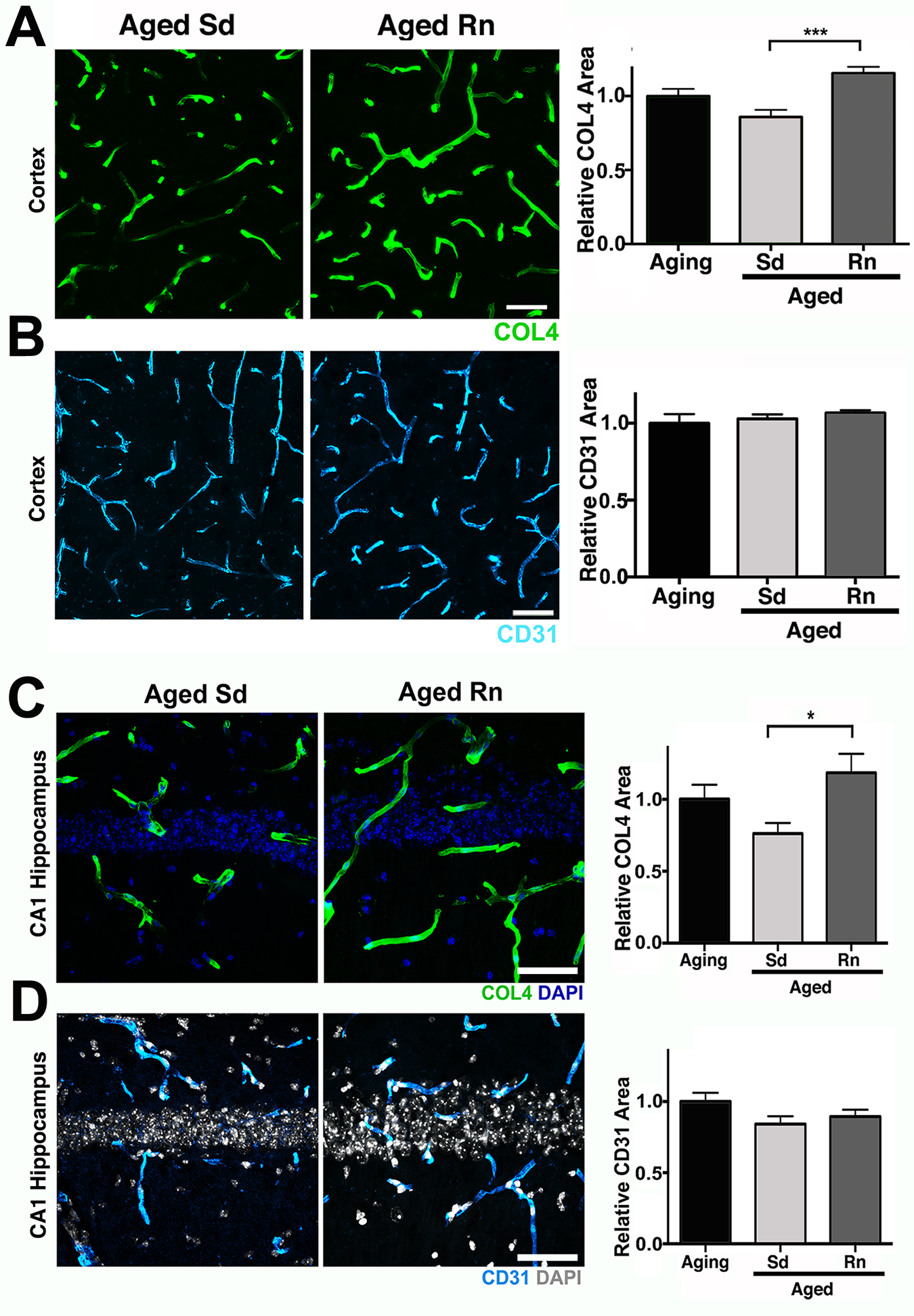

Supplement: S5 Fig — (A) COL4+ microvessels are significantly increased in the cortex of aged runner mice when compared with aged sedentary mice. (B) Quantification of CD31+ capillary area shows no significant differences between groups. (C) COL4+ microvessels are significantly increased in the CA1of aged runner mice when compared with aged sedentary mice. (D) Quantification of CD31+ capillary area in the CA1 shows no significant differences between groups. In (B–D), values are relative mean + SEM to the young values, n = 4 aging mice, n = 6 aged sedentary mice and n = 6 aged runner mice. In (A) ***p = 0.0007 and in (C) *p = 0.0163 by ANOVA followed by Tukey’s posthoc tests. Scale Bars: 50 μm. The data used to make this figure can be found in S1 Dataset. (TIF) [file pbio.1002279.s006.tif]

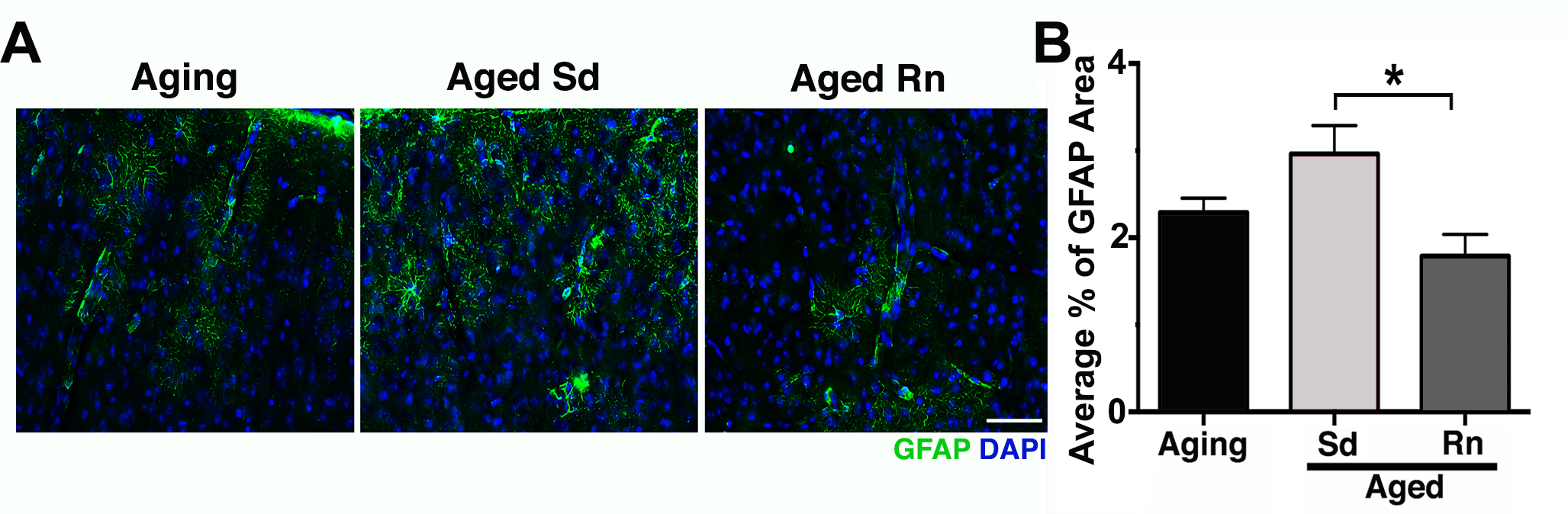

Supplement: S6 Fig — (A) Increased GFAP immunoreactivity in the cortex of aged sedentary mice is prevented by voluntary running in aged runner mice. (B) Quantification of GFAP+ area in the cortex of aging, aged sedentary and aged runner mice. Values are mean + SEM of the % area immunolabeled with GFAP, n = 4 mice per group. *p < 0.05 by ANOVA followed by Tukey’s posthoc tests. Scale Bar: 50 μm. The data used to make this figure can be found in S1 Dataset. (TIF) [file pbio.1002279.s007.tif]

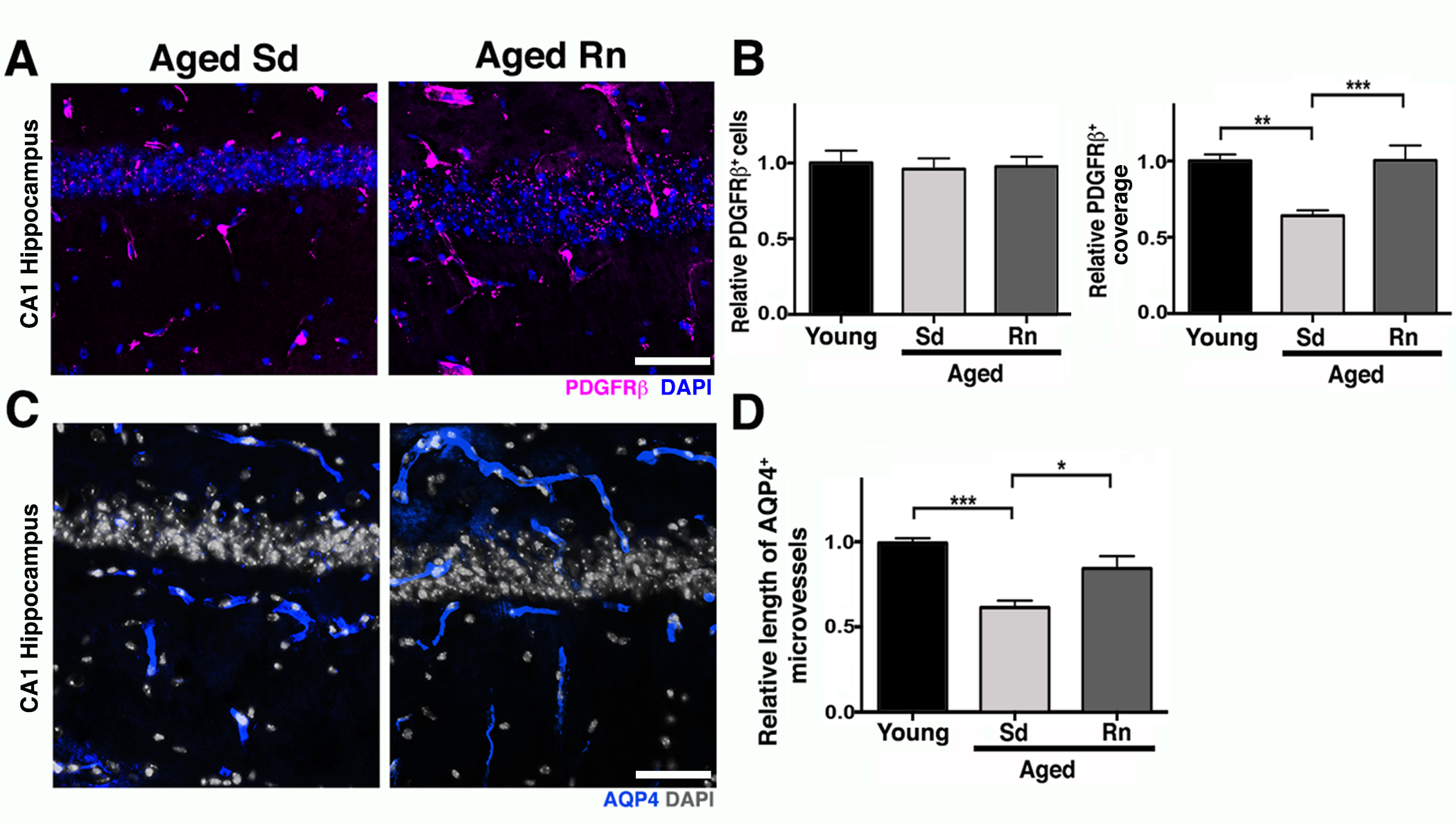

Supplement: S7 Fig — (A–B) PDGFRβ+ pericyte coverage is significantly increased in the CA1 region of aged runner mice when compared with aged sedentary mice. No changes in the number of PDGFRβ+ pericyte were found between groups. (C–D) AQP4+ microvessels are significantly increased in the CA1of aged runner mice when compared with aged sedentary mice. In (B and D), values are relative mean + SEM to the young values, n = 4 aging mice, n = 6 aged sedentary mice, and n = 6 aged runner mice. In (B) **p = 0.0011 and ***p = 0.0010 and in (D) ***p = 0.0009 and *p = 0.0183 by ANOVA followed by Tukey’s posthoc tests. Scale Bars: 50 μm. The data used to make this figure can be found in S1 Dataset. (TIF) [file pbio.1002279.s008.tif]

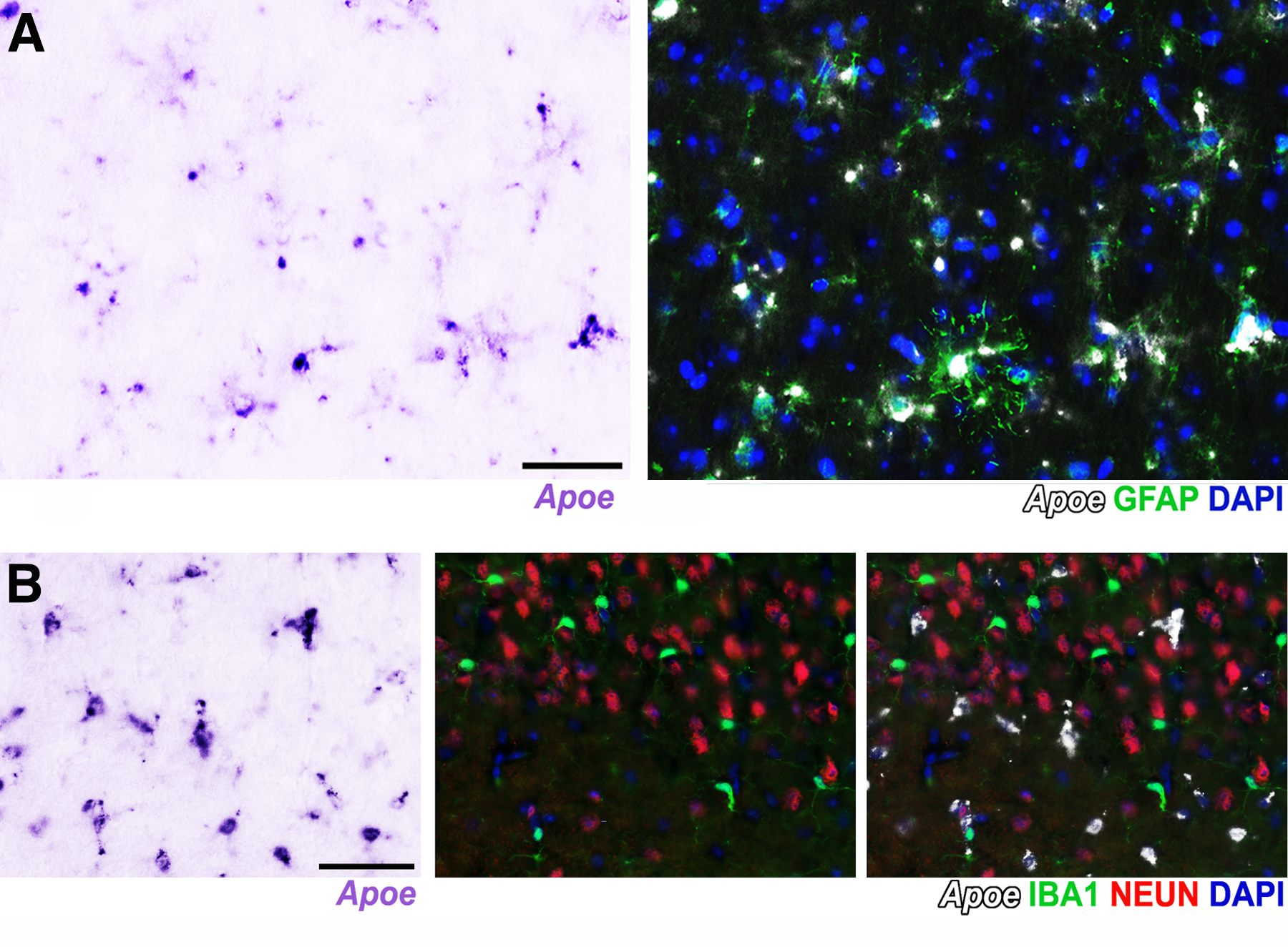

Supplement: S8 Fig — (A) Apoe in situ hybridization signal (purple/white) colocalized with GFAP immunostaining (green) in astrocytes. (B) Apoe expression (purple/white) is absent on IBA1+ microglia and NEUN+ neurons. Scale Bars: 50 μm. (TIF) [file pbio.1002279.s009.tif]

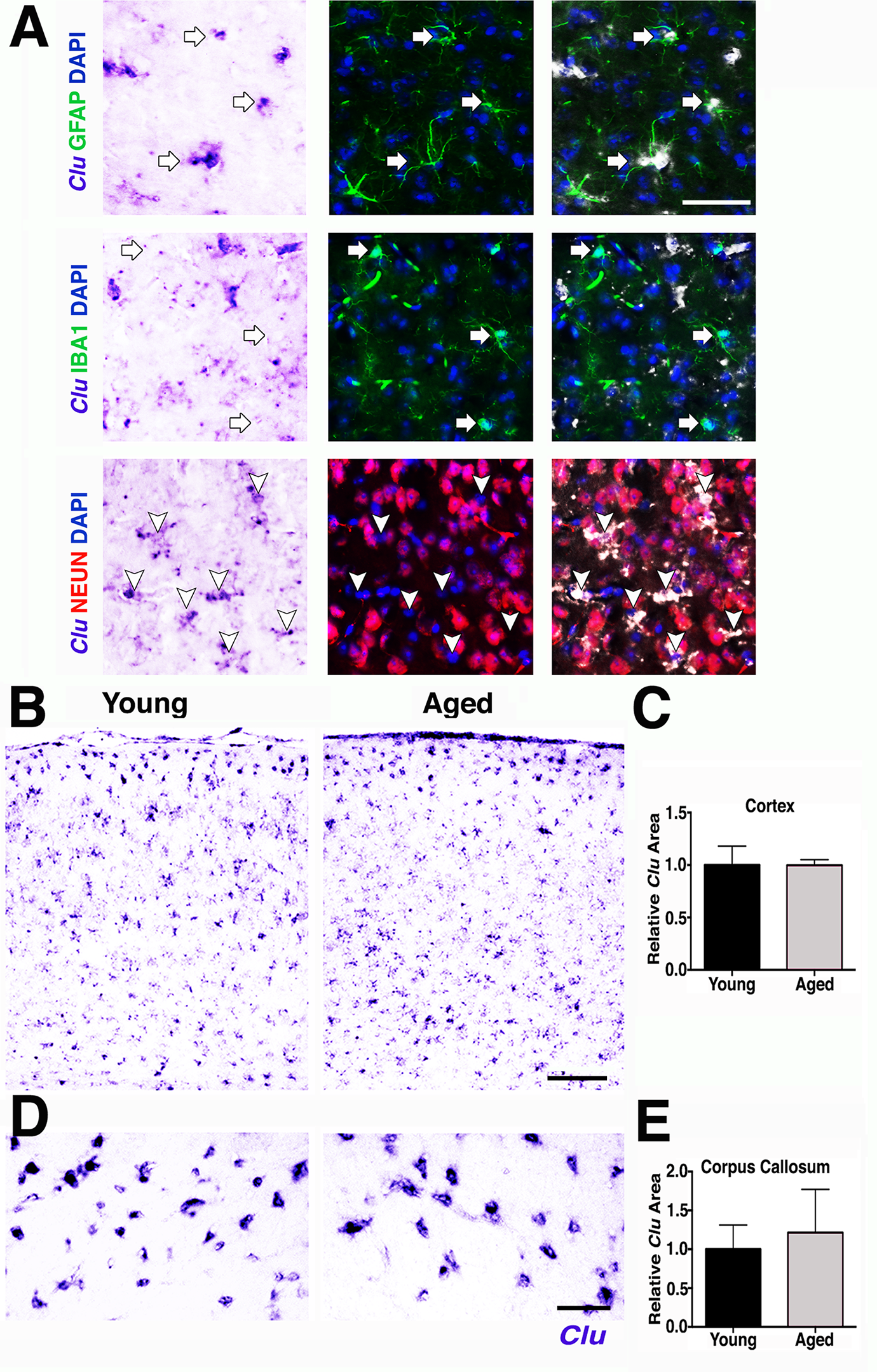

Supplement: S9 Fig — (A) Clu in situ hybridization signal (purple/white) colocalized with GFAP (green/astrocytes), IBA1 (green/microglia) and NEUN (red/neurons) immunostaining. Arrows indicate colocalization of GFAP with Clu expressing cells, but not IBA1+ microglial cells. Arrowheads show cells positive for Clu signal are negative for NEUN immunostaining. (B–C) Clu expression (purple) does not change with age in the neocortex. (D–E) No changes in Clu expression are found in the CC. In (C and E), values are relative mean + SEM to the young values, n = 4 mice per group. Scale Bars: 50 μm (A and D), 100 μm (B). The data used to make this figure can be found in S1 Dataset. (TIF) [file pbio.1002279.s010.tif]

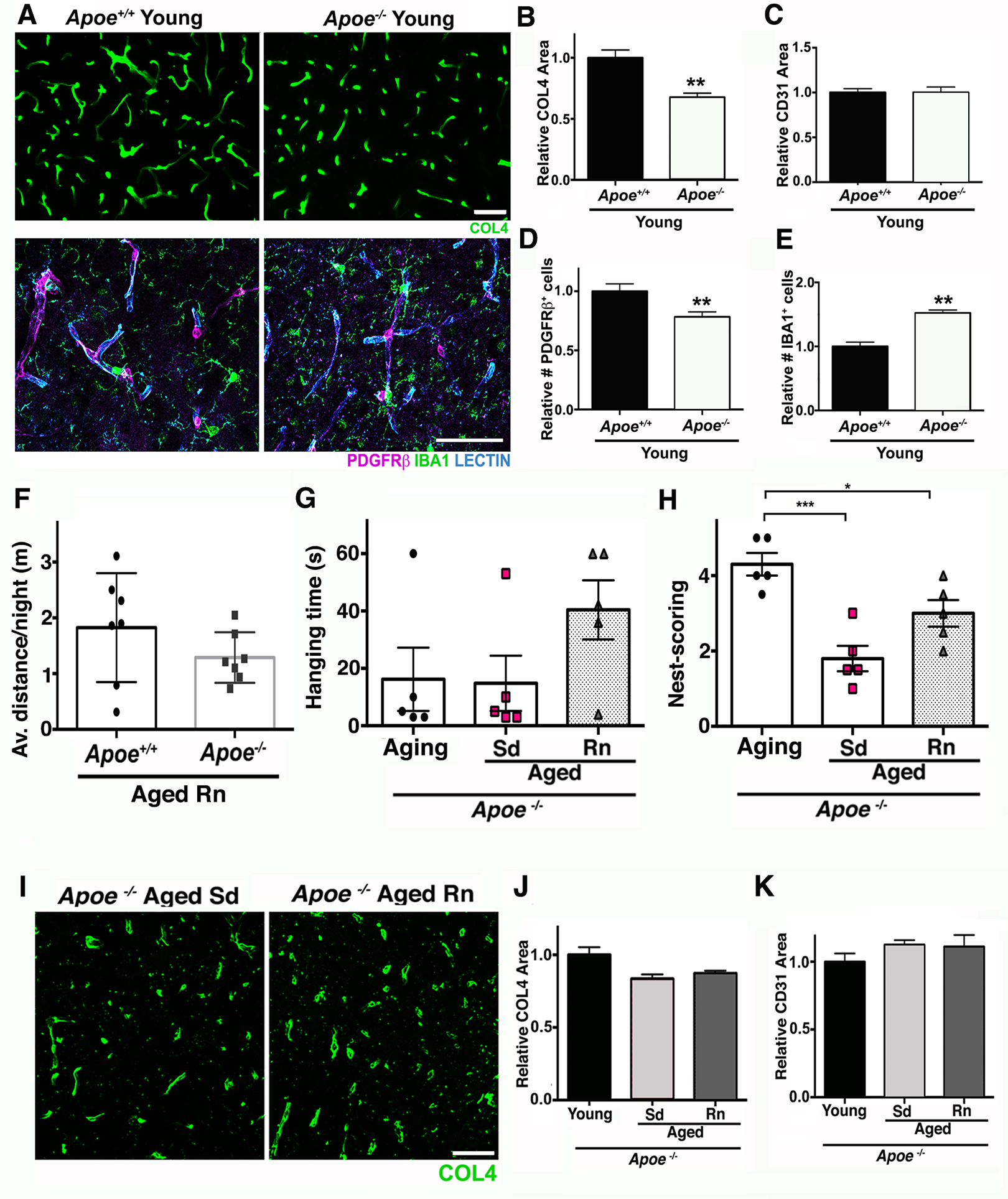

Supplement: S10 Fig — (A) Col4+ capillaries are significantly reduced on APOE-deficient mice. In (A) lower panel, merge images showing PDGFRβ+ pericytes (magenta), IBA1+ microglia (green) and LECTIN+ endothelial cells in the young Apoe +/+ and Apoe -/- mice. (B) Quantification of COL4+ capillary area between young (9 mo) Apoe +/+ and Apoe -/- B6 mice. (C) Quantification of CD31+ capillary area, nonsignificant changes are observed. (D) Quantification of PDGFRβ+ pericytes showing a significant decrease of these cells in the APOE-deficient mice. (E) Quantification of IBA1+ microglia showing a significant increase of these cells in the APOE-deficient mice. (F) Running distances by Apoe -/—aged mice were not statistically significant from Apoe +/+-aged runner mice. (G) Deficits in grip strength found in Apoe -/- aging and aged sedentary mice were not statistically significant from Apoe -/- aged runner mice, although a partial increase is observed. (H) Deficits in nest construction behavior were not preserved in Apoe -/—aged running mice when compared with Apoe -/—aged sedentary mice. (I–J) No changes in COL4+ capillary area were found between aged sedentary Apoe -/- and aged runner Apoe -/- mice. (K) Quantification of CD31+ capillary area in young Apoe -/-, aged sedentary Apoe -/- and aged runner Apoe -/- mice. In panels (B, D, E, F–H, J, and K) values are mean + SEM. In (B, D, and E) **p < 0.005, n = 4 by unpaired t test. In (H) *p = 0.0416 and ***p = 0.0005 by ANOVA followed by Tukey’s posthoc tests. Scale Bars: 50 μm. The data used to make this figure can be found in S1 Dataset. (TIF) [file pbio.1002279.s011.tif]

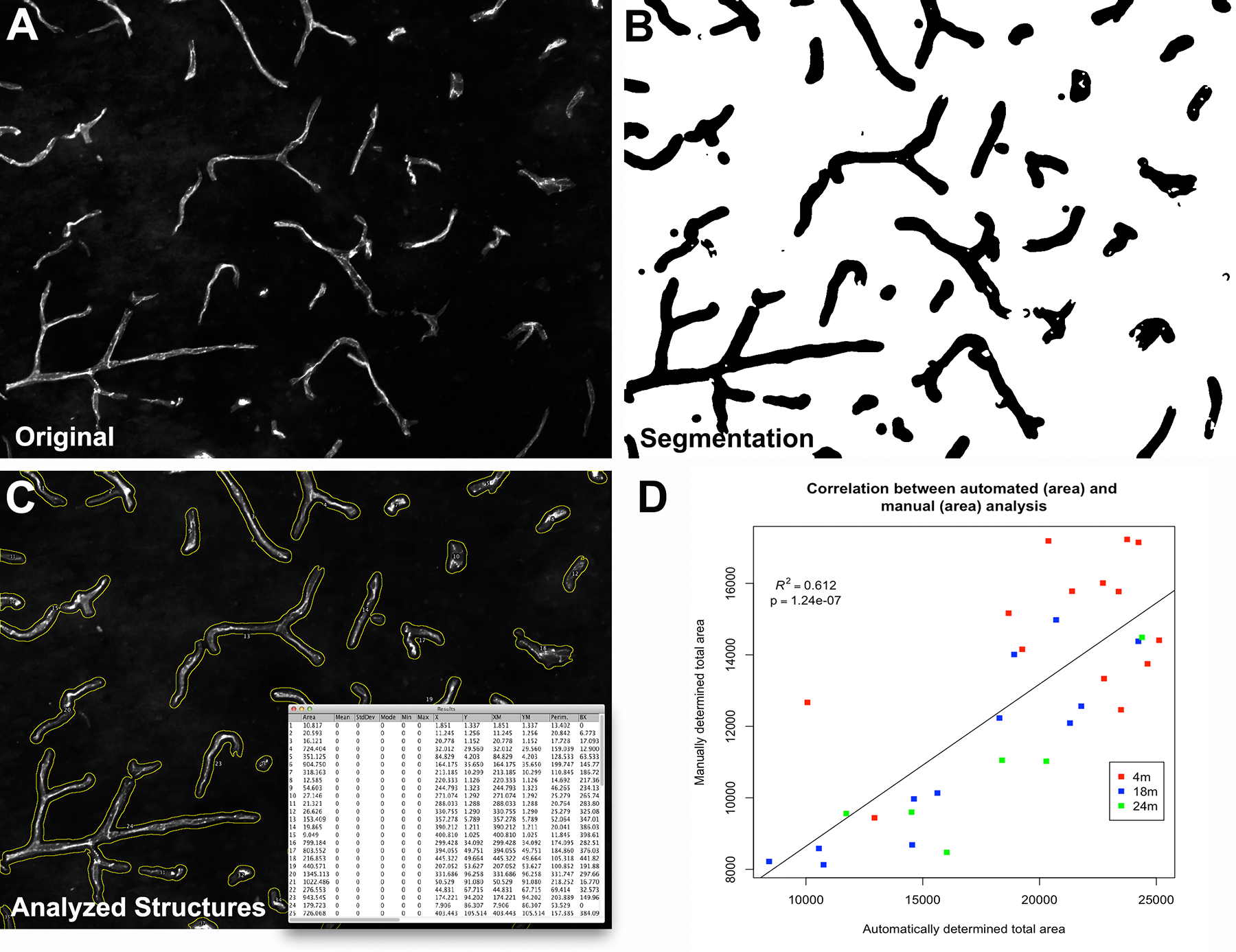

Supplement: S11 Fig — (A–C) Sample images showing steps of image-automated processing for microvessels area quantification. Original image show in A, invert step after segmentation show in B and analyzed structures in C. Inset on C shows the tabulated data acquired after the analysis. (D) A positive correlation (R2 = 0.612, p = 1.24e-07) is observed between vascular area calculation by manual segmentation versus automated segmentation by the segmentation algorithm developed in our lab. (TIF) [file pbio.1002279.s012.tif]
